# Genome-wide meta-analysis of brain volume identifies genomic loci and genes shared with intelligence

**DOI:** 10.1038/s41467-020-19378-5

**Published:** 2020-11-05

**Authors:** Philip R. Jansen, Mats Nagel, Kyoko Watanabe, Yongbin Wei, Jeanne E. Savage, Christiaan A. de Leeuw, Martijn P. van den Heuvel, Sophie van der Sluis, Danielle Posthuma

**Affiliations:** 1 Department of Complex Trait Genetics, Center for Neurogenomics and Cognitive Research, Amsterdam Neuroscience, Vrije Universiteit Amsterdam, Amsterdam, The Netherlands; 2 Department of Clinical Genetics, Amsterdam UMC, Vrije Universiteit Amsterdam, Amsterdam, The Netherlands; 3 Department of Child and Adolescent Psychiatry and Psychology, Section Complex Trait Genetics, Amsterdam Neuroscience, Vrije Universiteit Medical Center, Amsterdam UMC, Amsterdam, The Netherlands

**Keywords:** Behavioural genetics, Genome-wide association studies

## Abstract

The phenotypic correlation between human intelligence and brain volume (BV) is considerable (*r* ≈ 0.40), and has been shown to be due to shared genetic factors. To further examine specific genetic factors driving this correlation, we present genomic analyses of the genetic overlap between intelligence and BV using genome-wide association study (GWAS) results. First, we conduct a large BV GWAS meta-analysis (N = 47,316 individuals), followed by functional annotation and gene-mapping. We identify 18 genomic loci (14 not previously associated), implicating 343 genes (270 not previously associated) and 18 biological pathways for BV. Second, we use an existing GWAS for intelligence (N = 269,867 individuals), and estimate the genetic correlation (*r*_*g*_) between BV and intelligence to be 0.24. We show that the *r*_*g*_ is partly attributable to physical overlap of GWAS hits in 5 genomic loci. We identify 92 shared genes between BV and intelligence, which are mainly involved in signaling pathways regulating cell growth. Out of these 92, we prioritize 32 that are most likely to have functional impact. These results provide information on the genetics of BV and provide biological insight into BV’s shared genetic etiology with intelligence.

## Abstract (web summary)

Brain volume and intelligence have been previously found to have shared genetic etiology, but the specific common genetic signals have not been identified. Here, the authors perform a genome-wide association study on brain volume, finding common genetic loci driving brain volume and intelligence.

## Introduction

The relation between brain volume (BV) and cognitive ability has long been a fundamental question to cognitive sciences^1–4^. Before the advent of magnetic resonance imaging (MRI), BV was typically determined from autopsy records^5^, or inferred by external head size measures. A literature meta-analysis conducted by Vernon et al.^6^ estimated the correlation between external head measures and intelligence to be around 0.19. Five years later, a meta-analysis of 24 studies using MRI-based measures of BV (with the subtle title; “Big brains are better”) found a substantially higher correlation of 0.33 with intelligence^7^. Recently, an exhaustive meta-analysis^8^, including 88 studies comprising 148 samples, was conducted, aiming to readdress the much-debated relation between BV and intelligence. This study found that BV and intelligence were, indeed, robustly correlated, albeit lower (*r* = 0.24) than what was reported in the literature. The difference may in part be explained by publication bias, with strong, positive correlations more often being reported than small, non-significant ones. Recently, these findings were supported by another large-scale study reporting a correlation of *r* = 0.19 between BV and fluid intelligence^9^ (referring to problem-solving and logical skills that do not depend on previously acquired knowledge). Interestingly, previous studies did not find evidence for sex differences in the relation between BV and intelligence^8^.

The conservative correlation of ~0.20, implies an explained variance of just 4%. This is low and therefore BV is not a good predictor of intelligence, and vice versa, intelligence is not a good predictor of BV. However, knowing that this correlation is at least partly due to shared genetic factors^10^ suggests that knowledge of which specific genetic factors are involved in both traits may provide insight into the observed covariance between BV and intelligence. With the availability of larger genotype data and genome-wide association studies (GWAS) that identify increasing numbers of genes related to BV^11–13^ and intelligence^14–16^, analysis of the shared genes and pathways has become feasible.

In the current study, we utilize large-scale genetic data and advanced statistical genetics tools, to examine the genetic factors that underlie the observed correlation between BV and intelligence. We conduct a GWAS of BV in the UK Biobank (UKB) sample, and meta-analyze the results with two additional cohorts for which data on intracranial volume (ICV) and head circumference (HC), a proxy measure for BV, is available in the public domain. Using extensive follow-up analyses and GWAS summary statistics of intelligence we aim to zoom in on the genetic factors overlapping between BV and intelligence.

We identify 92 genes affecting both BV and intelligence, which are mainly involved in signaling pathways regulating cell growth. A series of follow-up analyses prioritizes 32 out of these 92 genes that are most likely to have functional impact based on the properties of these genes.

## Results

### GWAS meta-analysis of BV

**Fig. 1:**
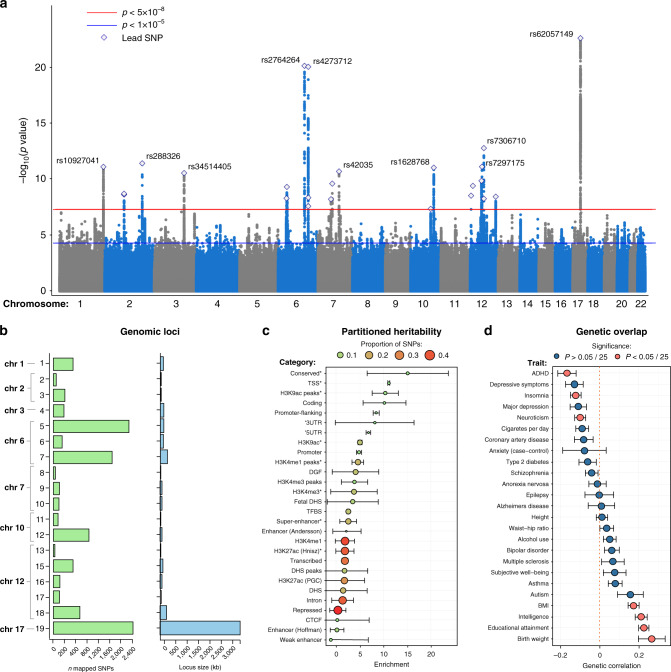
Genome-wide meta-analysis and follow-up analyses of brain volume (BV). **a** Manhattan plot of the GWAS meta-analysis of BV in 47,316 individuals showing the genomic position of each variant on the *x*-axis and the meta-analysis negative log_10_-transformed *P-*value on the *y*-axis. Independent lead variants of each locus are annotated by a diamond. The horizontal red line indicates the genome-wide significance threshold that corresponds to a *P*-value of 5 × 10^−8^, while the horizontal blue indicates the suggestive threshold of 1 × 10^−5^. **b** Overview of the genomic loci sizes and number of variants mapped by each locus. Note that we detected 19 loci, but report 18, as we were less confident in one locus (see Supplementary results 2.1). **c** Partitioning of the SNP heritability of the BV GWAS meta-analysis by binary variant annotations using LD Score regression. Enrichment was calculated by dividing the partial heritability of a category by the proportion of variants in that category (proportion indicated by color and size of the dots). Significant enrichments, corrected for the number of categories tested (*P* < 0.05/28), are highlighted with an asterisk. **d** Genetic correlations with previous traits estimated using LD Score regression. Red dots denote significant genetic correlation after Bonferroni correction (*P* < 0.05/25), the vertical dashed line represents a genetic correlation of 0. Error bars represent standard errors of the estimates.

To identify genetic variants associated with BV, we first performed a GWAS (Supplementary Fig. 1) using data of 17,062 participants from the UKB^17^, with BV estimated from structural (T_1_-weighted) MRI by summing total gray and white matter volume, and ventricular cerebrospinal fluid volume (Supplementary Fig. 2). GWAS analyses in UKB were corrected for the Townsend deprivation index (TDI)^18^, a measure that correlates with socioeconomic and health-related factors^19,20^, as well as for age, sex, genotype array, assessment center, standing height, and the first 10 genetic principal components (PCs). Within the UKB data, we identified 3,610 genome-wide significant (GWS; *P* < 5 × 10^−8^) variants, tagging 9 independent genomic loci. The single nucleotide polymorphism (SNP)-based heritability (*h*^*2*^_SNP_) of BV, estimated through linkage disequilibrium score regression (LDSC^21^; Methods), was 35.3% (SE = 4.1%). The LDSC intercept of 1.02 is close to 1, suggesting that the observed inflation (*λ*_GC_ = 1.118) in genetic signal is mostly due to polygenicity rather than population stratification^22^ (see Supplementary Note 1). The UKB GWAS results were then meta-analyzed with GWAS results from two previously published studies: one on ICV from the ENIGMA consortium^13^ (*N* = 11,373), the other on HC, a proven proxy of BV^23–25^ (also see Supplementary Note 2), from a recent GWAS meta-analysis^11^ of adults and children (*N* = 18,881). This led to a total sample size of 47,316 unrelated Europeans (Supplementary Fig. 3; Supplementary Table 1). LDSC showed high concordance of SNP associations between the three samples (UKB and ENIGMA: *r*_*g*_ = 1.25, SE = 0.20; UKB and HC-GWAS: *r*_*g*_ = 0.75, SE = 0.09; ENIGMA & HC-GWAS: *r*_*g*_ = 0.94, SE = 0.20; Supplementary Data 1), justifying subsequent meta-analysis (we note that the *r*_*g*_ outside [−1, 1] is likely due to the unbiased *r*_*g*_ estimation by LDSC, the large standard error of the estimate and the high genetic correlations between these meta-analyzed cohorts). Sample-size weighted fixed-effects meta-analysis was carried out using METAL^26^ (Methods) resulting in 24 linkage disequilibrium (LD) independent lead variants (*r*^*2*^ < 0.1), residing in 18 genomic loci (Fig. 1a–b; Supplementary Data 2; Supplementary Figure 4; Supplementary Note 3), representing 4,155 GWS variants (of which 371 were indels, Supplementary Data 3) associated with BV. Of these 18 loci, 14 were not identified in a recent GWAS study of ICV^12^ (2 loci reported in this earlier study were not GWS in the current meta-analysis). To see if our results were driven by only one of the three samples or were supported by all three, we examined the direction of effect of all GWS variants across the three individual cohorts. As expected, we found high concordance: all variants that were GWS in the meta-analysis, and that were present in all three cohorts, had the same direction of effect. Of the 4,155 variants that were GWS in the BV meta-analysis, just 2.4% was GWS in at least two of the individual cohorts, however, 75.0% had a *P*-value < 0.05 in at least two cohorts (and 21.2% in all three cohorts). The *h*^*2*^_SNP_ of BV estimated by LDSC from the meta-analytic results was 21.4% (SE = 1.8%), and the LDSC intercept approximated 1 (1.025, SE = 0.009), suggesting that the inflation in test statistics (*λ*_GC_ = 1.18) in the meta-analysis was also largely due to polygenicity^22^. Functional annotation of 4,683 ‘candidate’ variants (i.e., variants in the loci with a GWAS *P-*value of *P* < 10^−5^ and LD *r*^*2*^ > 0.6 with one of the independent significant variants; Methods; Supplementary Note 4) carried out in FUMA^27^ showed that these variants were most abundant in intronic (*n* = 2,444, 52,1%) or intergenic regions (*n* = 751, 16.0%), and 27 (0.6%) variants were exonic nonsynonymous SNPs (ExNS) altering protein structures of 13 genes (Supplementary Figure 5; Supplementary Data 4, 5). One gene, *SPPL2C*, contained 8 ExNS (all in exon 1, and in the same inversion region). *SPPL2C* codes for the signal peptide peptidase-like 2C, which plays a role in the degradation of signaling peptides in the brain^28^.

To test whether specific functional categories of variants contribute disproportionally to the heritability of BV, we used LDSC to partition the genetic signal over different variant annotations^29^ (Methods). We observed significant heritability enrichment in eight variant categories (Fig. 1c; Supplementary Table 2), with the strongest enrichment of variants in (evolutionary) conserved regions, (enrichment = 15.1, SE = 2.7, *P* = 1.19 × 10^−6^, suggesting a 15-fold enrichment in *h*^2^ conveyed by variants in these regions compared to the proportion of variants in these regions), transcription start sites (TSS; enrichment=11.1, SE = 3.1, *P* = 1.2 × 10^−3^), and H3K9ac peaks (i.e., specific histone modification that is correlated with active promoters; enrichment = 10.3, SE = 3.0, *P* = 1.24 × 10^−3^).

### BV gene analyses

**Fig. 2:**
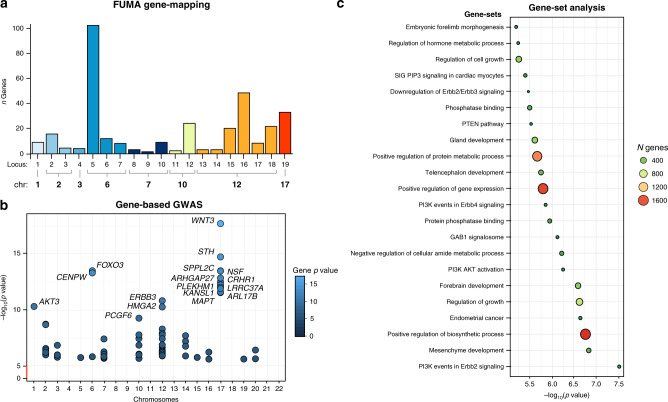
Gene analyses based on the GWAS meta-analysis of BV. **a** Number of genes that were mapped by FUMA to each locus. Colors indicate distinct chromosome numbers. **b** Significant genes in the gene-based association test in MAGMA after Bonferroni correction (*P* < 0.05/18,169). The *x*-axis represents the chromosome number, *y*-axis and the dot color shows the negative log_10_-transformed gene-based *P-*value. The top 15 most significant genes are annotated with the corresponding gene symbols. **c** Significant MsigDB gene sets after Bonferroni correction (*P* < 3.90 × 10^−6^ (=0.05/(12,191 + 53 + 565)) showing the negative log_10_-transformed *P-*value on the *x*-axis; size and colors of the circles represent the size of the gene set.

To gain insight into which genes may be involved in BV, we mapped the 4683 candidate variants implicated in the BV meta-analysis to genes, using positional mapping, eQTL mapping, and chromatin interaction mapping as implemented in FUMA^27^, and through gene-based association tests as implemented in MAGMA^30^ (Methods, Supplementary Fig. 6; Supplementary Note 5). In total, 343 unique genes were implicated by at least one of these methods (of these, 321 genes were mapped based on the 18 identified risk loci). Specifically, the 18 risk loci were mapped to 119 genes based on position, 207 genes by eQTL association, 192 genes through chromatin-chromatin interactions (Fig. 2a; Supplementary Data 6), and gene-based association testing identified 69 genes (Fig. 2b; Supplementary Data 7). Of the 343 genes, 73 genes were also found in previous studies (38 were not), while 270 were not previously implicated (Supplementary Data 8). Overall, 16 genes were implicated by all four methods (*FRZB*, *FOXO3*, *WBP1L*, *PRR13*, *MAP3K12*, *RAB5B*, *SUOX*, *RPS26*, *ERBB3*, *PITPNM2*, *C12orf65*, *INA, HMGA2, PLEKHM1*, *MAPT*, and *KANSL1*) of which the first 11 were not identified in previous studies of BV at the time of analysis^11,12,31–33^.

Variation in BV can be seen as a continuum, with abnormally small or large brains as the extremes. Since monogenic Mendelian disorders are often characterized by abnormal brain development, which frequently results in deviations in BV, we performed look-up of the 343 genes implicated in the BV meta-analysis in the Online Mendelian Inheritance in Man database^34^ (OMIM). We identified 143 monogenic disorders caused by high-penetrance mutations in 89 of the 343 BV-implicated genes (25.9% vs. 16.4% (59 genes) that is expected based on the number of genes reported in OMIM; Fisher exact test *P* = 2.33 × 10^−5^) many of which are commonly associated with abnormal brain development (including microcephalia (*CDK6*), macrocephalia (*PTEN*) and megalencephaly (*AKT3*)), and abnormal growth (tetra-amelia syndrome (*WNT3*)) (Supplementary Data 9).

To identify functional pathways related to BV, we performed gene-set and gene-expression analysis in MAGMA^30^ (Methods). Of the 12,191 tested gene sets (including canonical pathways, gene ontology (GO) gene sets, and gene-expression in tissue/cell types), 18 were significantly associated after Bonferroni correction (Gene-set *P*_BONF_ = 3.90 × 10^−6^ (0.05/(12,191 gene sets + 53 tissue types + 565 cell types)); Fig. 2c; Supplementary Data 10–11; Supplementary Note 6). Among these significant gene sets were several cell-signaling pathways, including the *ERBB3* pathway, the PI3K/AKT signaling in cancer pathway and a pathway related to *GRB7* events in ERBB2 signaling. Other pathways were related to (regulation of) developmental processes such as mesenchyme and muscle organ development, or to growth. That is, key cell-signaling pathways were implicated that are involved in normal brain development^35^ and involved in several brain developmental abnormalities^36,37^. Pairwise conditional gene set analysis allows one to disentangle overlapping associations of gene sets (see Methods). Conditional gene-set analyses can be used to examine whether a gene set is independently associated with a trait, rather than being associated because it is nested within a larger associated gene set^38^. These analyses indicated that nine gene sets constitute largely independent associations, defined as remaining significant in the majority of the conditional analyses (Supplementary Figs. 7–9, Supplementary Data 12). Conversely, conditional analyses suggest that a number of signaling pathways (BIOCARTA: ERBB3 pathway, BIOCARTA: PTEN pathway) represent a shared underlying signal, since conditional *P*-values were substantially higher than the unconditional *P*-values.

Next, we aimed to identify tissue categories and neuronal cell types that are enriched for gene signal of BV, by linking gene *P-*values to gene-expression in 53 tissue types^39^ and 565 brain cell types^40^ (Methods). None of the associations of tissue or cell types passed our stringent multiple testing correction (i.e. correcting for all tested gene sets, tissues, and cell types, thus 0.05/12,809). The strongest evidence of association was observed for three cell types: *TNR-BMP4* polydendrocytes in the subthalamic nucleus, (*P* = 4.67 × 10^−3^), layer 5a cortical (*P* = 8.55 × 10^−3^), and hippocampal endothelial stalk *FLT1-LCN2* neurons (*P* = 0.01) (Supplementary Data 10). Polydendrocytes are thought to be precursor cells of oligodendrocytes^41^, a cell-type involved in supporting neuronal health and myelinization of the brain^42^. The endothelial neurons in the hippocampus support formation of the blood–brain barrier, and are involved in the regulation of neurogenesis^43^.

### Genetic correlations between BV and other traits

Earlier studies have shown substantial overlap between BV and behavioral traits, reporting phenotypic correlations between BV and, e.g., autism^44^, and genetic correlations between BV and intelligence^45–48^ and schizophrenia^49^. We used LDSC to estimate the overlap in genetic signal between BV and 25 brain-related and neuropsychiatric traits for which published genome-wide summary statistics based on large samples were available (Methods). Significant genetic correlations (*P* < 0.002; 0.05/25) were observed between BV and 7 traits, amongst which were positive genetic correlations with educational attainment (*r*_*g*_ = 0.22, SE = 0.03, *P* = 3.58 × 10^−17^; Fig. 1d; Supplementary Table 3), intelligence (*r*_*g*_ = 0.21, SE = 0.03, *P* = 9.87 × 10^−12^), BMI (*r*_*g*_ = 0.17, SE = 0.03, *P* = 1.22 × 10^−9^) and birth weight (*r*_*g*_ = 0.26, SE = 0.07, *P* = 1.13 × 10^−4^), and negative genetic correlations with ADHD (*r*_*g*_ = −0.17, SE = 0.05, *P* = 3.56 × 10^−4^), neuroticism (*r*_*g*_ = −0.10, SE = 0.03, *P* = 4.65 × 10^−4^), and insomnia (*r*_*g*_ = −0.12, SE = 0.03, *P* = 5.76 × 10^−6^), confirming previously reported overlap^12,50,51^

### Genetic overlap with intelligence

The development of human intelligence has coincided with a strong increase in total size of the brain^52^. Indeed, epidemiological studies have shown overlap in genetic factors between BV and intelligence^46,53^. To further explore the nature of the genetic overlap between intelligence and BV, we used GWAS summary statistics of a large recent meta-analysis of intelligence from Savage et al.^53^, based on data from 14 cohorts (*N* = 269,867) using different types of intelligence measures. Except for one, all of these cohorts used intelligence measures that adhere to the idea of a single latent trait *g* (Spearman’s *g*) that underlies multiple domains of cognitive ability. Extensive previous research has shown that a) scores on different, validated tests of cognitive ability correlate highly, and b) all tests index the same underlying (statistical) construct, i.e., *g*^10,54^.

**Fig. 3:**
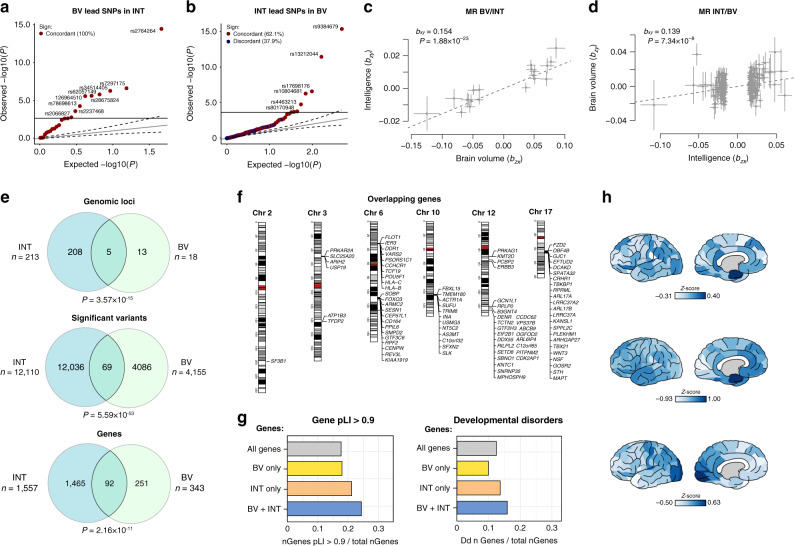
Analyses of the genetic overlap between brain volume and intelligence. **a** Q-Q plot showing the observed and expected *P-*value distribution of 24 lead variants of the brain volume (BV) meta-analysis in intelligence (INT) and **b** 243 intelligence meta-analysis lead variants in BV. The colors denote the concordance (/discordance) of effects between the two traits. **c** Mendelian Randomization analysis, showing the effect size and standard errors of each variant of BV (*b*_*zy*_) on intelligence (*b*_*zx*_) and the diagonal line showing the estimated causal effect (*b*_*xy*_) of BV on intelligence. **d** Similar to (**c**) but of intelligence on BV. **e** Venn diagrams showing the number of overlapping loci, significant variants, and genes implicated in BV and intelligence by FUMA and MAGMA. **f** Karyogram showing the genomic position of the 92 genes that were implicated in the GWAS of both BV and intelligence. **g** Comparison of the pLI score (representing tolerance to loss-of-function variants) and fraction of genes involved in developmental disorders (DECIPHER database) between BV-only genes, intelligence-only genes (INT), genes associated to both traits (BV + INT), and all genes. **h** Cortical gene expression patterns (darker colors indicate higher rank scores) across 57 cortical areas of the 92 genes that overlap between BV and intelligence (top panel), and the two clusters of genes (middle and bottom panel).

Our analyses confirmed the previously established genetic overlap between BV and intelligence: the estimated *r*_*g*_ between BV and intelligence was 0.24 (Supplementary Data 2; Supplementary Table 3), in line with prior evidence of shared genetic factors between intelligence and gray and white matter volume obtained from twin studies^46^. To gain insight into the specific genetic factors driving this genetic correlation, we explored the overlap in variants, loci, and genes associated with both traits. We first determined whether strongly associated variants show the same direction of effect in both traits, by performing a look-up of the lead variant of BV in the intelligence GWAS, and vice versa. We observed a complete sign concordance of the 24 BV lead variants in intelligence (sign concordance = 100%, *P* = 1.19 × 10^−7^, Fig. 3a), and weaker but still considerable sign concordance of the 243 lead variants of intelligence in BV (sign concordance = 62.1%, *P* = 1.39 × 10^−4^, Fig. 3b). Similarly, we found that the majority of genes that reached gene-wide significance in the MAGMA gene-based test of BV (69) also showed low *P-*values in intelligence (507), and vice versa (Supplementary Fig. 10).

To explore possible causal relations between BV and intelligence, we carried out GWAS summary statistics-based Mendelian Randomization (MR) analyses, using the Generalized Summary-data-based MR package^55^ (GSMR; Methods), essentially using independent lead variants as instrumental variables (LD: *r*^*2*^ < 0.1). GSMR analyses demonstrated a directional effect of BV on intelligence (*b*_*xy*_ = 0.154, SE = 0.015, *P* = 1.88 × 10^−23^; Fig. 3c) and a less strong yet still highly significant directional effect of intelligence on BV (*b*_*xy*_ = 0.139, SE = 0.026, *P* = 7.34 × 10^−8^; Fig. 3d), suggesting a bidirectional association between these phenotypes, in line with the previous reports^53^. We do note, however, that GSMR relies on several strong assumptions, such as the absence of a third, mediating, factor, and no horizontal or vertical pleiotropy^56^, which may not always hold. The current results should be interpreted conditional on these assumptions.

To investigate whether specific variants or genes could be identified that drive the genetic overlap between intelligence and BV, we performed several cross-trait analyses of variants and genes significantly implicated in both traits. We observed physical overlap in 5 out of the 18 genomic loci for BV, and overlap in genes implicated by FUMA (*n*_genes_ = 81) and MAGMA (*n*_genes_ = 24), resulting in 92 unique overlapping genes (Fig. 3e–f; Supplementary Data 13). Conversely, of the 343 genes that were significant for BV, 251 genes were not associated to intelligence (Fig. 3e). Lookup of gene functions of the 92 overlapping genes in the online GeneCards^57^ repository showed strong involvement of these genes in a wide variety of cellular processes and key factors in cell division. When comparing the probability of being loss-of-function (LoF) intolerant (pLI score >0.9) between overlapping genes (*n* = 92) and genes observed for only one of these traits (*n* = 1926), we observe a slightly higher fraction of genes being LoF intolerant in the genes that play a role in both traits (24.4% (shared) vs. 21.2% (intelligence-only), 18.0% (BV-only), and 17.7% (all genes), Fig. 3g). The mutation intolerance of these overlapping genes is further demonstrated by a slightly higher fraction of genes in this category that are associated with monogenic disease (18.3%) in the OMIM database compared to BV-only (16.8%) and intelligence-only (17.4%) genes (all genes: 16.4%). Furthermore, we observed a higher proportion of genes that cause monogenic developmental disorders (overlap: 16.0%, BV-only: 10.0%, intelligence-only: 13.7%, all genes: 12.3%) in the DECIPHER database of developmental disorders^58^ (Fig. 3g).

Previous studies have sought to narrow down the link between intelligence and BV to specific subcortical regions, such as the caudate nuclei^59^ or the thalamus^60^. If specific brain regions are related to intelligence, one might expect genes associated with intelligence (or BV) to be differentially expressed across regions. Using data obtained from the Allen Human Brain Atlas (AHBA)^61^, we examined whether cortical gene expression profiles of the 92 genes that overlap between BV and intelligence differed from the gene expression profiles of all the 1900 genes associated to BV and/or intelligence (Fig. 3h). Specifically, using permutation analyses we compared the expression of these 92 overlapping genes across 57 brain regions to that of 10,000 randomly selected, equally large, sets of genes drawn from the 1900 genes related to either or both of the traits. Although we observed overexpression of the 92 overlapping genes in the anterior part of the fusiform (two-sided permutation test *N* = 10,000 permutations *P* = 0.015) and the parahippocampal gyrus (*P* = 0.005), none of the associations survived a conservative Bonferroni correction (*P* = 8.77 × 10^−4^; 0.05/57); Supplementary Data 14). To examine whether clusters of more homogeneously expressed genes exist within the 92 overlapping genes, we additionally performed clustering analysis on the correlations between the expression profiles of genes, aiming to maximize intra-cluster cohesion, while at the same time maximizing differences between clusters (see Methods). Two clusters were identified and only for cluster 2 (representing 42 of the 92 overlapping genes) one of the 57 expression profiles was significantly different from that of random sets, suggesting overexpression in the anterior fusiform gyrus (*P* = 6.0 × 10^−4^; Supplementary Data 14). Interaction analyses in MAGMA (see Methods), testing whether gene expression in specific brain regions contributed significantly to the genetic relationship between BV and intelligence, revealed no evidence for interaction (Supplementary Data 15). Based on these results, we are unable to conclude that the set of overlapping genes is predominantly expressed in any particular cortical region compared to other brain regions.

To prioritize from the 92 overlapping genes those genes that are more likely to have causal effects on both BV and intelligence, we filtered the set of overlapping genes based on either one of three conditions: (1) the gene contains an ExNS SNP, (2) the gene is part of one of the significant gene sets for BV or intelligence, and (3) the GWAS signal of either trait colocalizes with eQTL signals, using COLOC^62^ (colocalization of GWAS and eQTL signals is compatible with the hypothesis of a common causal variant; Supplementary Data 13). Filtering the 92 overlapping genes on the basis of these 3 conditions yielded a selection of 32 potentially functionally interesting genes. For example, we prioritized two genes on chromosome 3 (out of 4 associated genes in the same region) that may prove particularly interesting candidates for functional follow-up aimed at characterizing the genetic relation between BV and intelligence: both *USP19* and *ARIH2* are part of a significantly associated gene set for BV, and in addition *USP19* contains an ExNS variant that was significantly associated to intelligence.

**Fig. 4:**
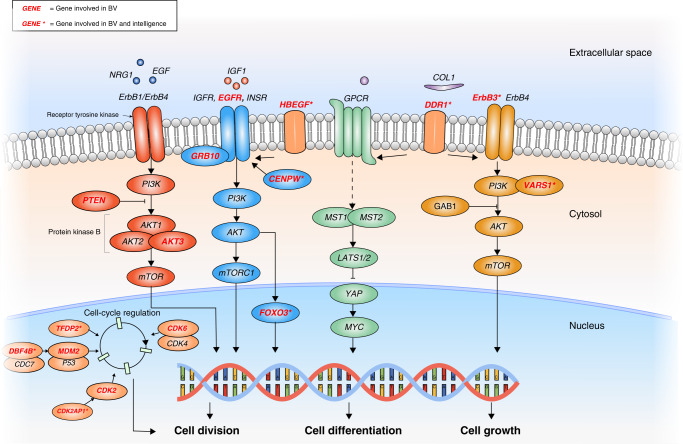
Schematic representation of cellular signaling pathways involved in BV. Overview of cellular signaling pathways and implicated genes within these pathways that were mapped by the GWAS meta-analysis of BV or overlapped with the genes that were mapped by the GWAS meta-analysis of intelligence. Genes in red font were involved in the meta-analysis of BV, whereas genes in red font denoted by an asterisk were observed in both the BV and intelligence GWAS meta-analyses.

To examine whether the gene-set associations are unique to BV and intelligence, respectively, we conducted conditional gene-set analyses (i.e., we conditioned the gene-based Z-scores in BV on the gene-based Z-scores for intelligence, and vice versa). Interestingly, these analyses indicated that the gene sets identified for BV (18) and intelligence (9) are trait specific, since the association remains virtually unchanged when conditioning on the other trait (Supplementary Table 4). Fisher’s exact tests showed that the 92 overlapping genes were not significantly enriched in the 27 gene sets associated to BV or intelligence (Supplementary Table 5). However, several genes related to intelligence were included in gene sets observed for BV, including *ERBB3* and *FOXO3* (see Fig. 4), suggesting that, although no single gene set was shared between BV and intelligence, several genes associated with intelligence are located along important signaling pathways implicated in BV.

## Discussion

We studied the genetics of BV and report 14 loci and 270 genes that had not been previously associated to BV. The gene-findings converged into 18 significantly associated gene-sets related to cellular signaling, division, and growth, mostly appearing independent in conditional analyses.

We found a significant positive genetic correlation with intelligence using previously published GWAS result^53^, confirming the genetic overlap estimated from twin studies^46^. We then explored whether specific genes or gene-sets drive this genetic correlation, and identified 92 genes that are associated with both traits. Of these 92, 32 genes were indicated as the most plausible ones explaining the shared genetic etiology. Several of these genes are involved in cell growth and differentiation pathways (e.g., *FOXO3, ERBB3, DDR,* and *HBEGF*, Fig. 4). We also tested whether the set of 92 shared genes, had specific biological characteristics that would set them apart from the genes that were associated with either BV or intelligence. Except for an increased intolerance to LoF mutations, we did not find any other of the tested biological functions to be different. We note that that the current set of genes associated with both traits, likely underestimates the true number of shared genes, as current sample sizes of especially the BV GWAS may limit the number of significantly associated genes.

The nature of the genetic overlap between intelligence BV remains largely unresolved. The large numbers of genes (>1000) that well-powered GWAS are now able to identify^53,63,64^ provide a starting point for understanding how genetic variation contributes to intelligence, yet it does not directly provide insight into the genetic relation with BV. Investigating genes that are related to both brain function (intelligence) and structure (BV), may provide further insight into the nature of the relation between intelligence and BV. We emphasize that in addition to shared pathways and biological pleiotropy, some genes linked to intelligence may also be associated to BV through gene-environment correlation with factors that indirectly influence BV development^65^.

In conclusion, we explored and refined the genetic architecture of BV, and identified and characterized a set of genes that likely drives this association.

## Methods

### Samples and phenotypes

UK Biobank - Brain volume: The UKB (UKB, www.ukbiobank.ac.uk) constitutes a large data set, combining a wide range of phenotypes with genetic and imaging information. The UKB study received ethical approval from the National Research Ethics Service Committee North West-Haydock (reference 11/NW/0382), and all study procedures were in accordance with the World Medical Association for medical research. The current study was conducted under UKB application number 16406. Here, we used processed data of a subset of *N* = 21,407 individuals who underwent a MRI procedure: data were released in the third quarter of 2018. After filtering on quality of the imaging results, relatedness, European ancestry and the availability of relevant covariates (discussed in more detail below), we arrived at a final sample size of *N* = 17,062. The BV phenotype was approximated as follows: BV = white matter volume + gray matter volume + cerebrospinal fluid volume (UKB field codes 25008, 25006, and 25004, respectively). The UKB obtained ethical approval from the National Research Ethics Service Committee North West–Haydock (reference 11/NW/0382), and all study procedures were performed in accordance with the World Medical Association for medical research. The current study was conducted under UKB application number 16406.

ENIGMA - ICV: We used GWAS summary statistics on ICV obtained in a collaboration of cohorts included in the meta-analysis of ICV of the Enhancing NeuroImaging Genetics through Meta-Analysis (ENIGMA)^13^ consortium. ICV was calculated from brain MRI data collected in 50 individual cohorts (*N* = 11,373). Given the moderate phenotypic and relatively low, but still significant, genetic correlation between BV and height (phenotypic *r* = 0.55 in UKB; *r*_*g*_ = 0.26 between height in UKB and uncorrected ICV summary statistics from ENIGMA^13^) we considered height an important covariate to include in GWAS on volumetric brain measures. Therefore, we used mtCOJO to correct for height, by conditioning on height using UKB-derived sumstats (this procedure is described in more detail below). Details on the individual cohorts included in the ENIGMA meta-analysis and genotyping, imputation and analysis procedures are described elsewhere^13^. All participants were of European descent and provided informed consent. All studies included in the ENIGMA meta-analysis were approved by their local institutional review board or local ethics committee.

HC GWAS: In contrast to the two samples discussed above, the GWAS summary statistics obtained from the HC GWAS are based on genetic analysis of an indirect measure of BV, i.e., HC in children and adults (see Supplementary Note 2 for more information on the relation between HC and BV). These summary statistics were generated by meta-analyzing 11 population-based cohorts, resulting in a total sample size of *N* = 18,881. All studies included in the meta-analysis were approved by the local ethics committees, and informed (or parental) consent was obtained from all participants. Data collection, genotyping, imputation and analysis procedures are described in detail elsewhere (see Haworth et al.^11^). Since not all individual studies that were included in the HC meta-analysis corrected for height, we first conditioned the HC GWAS summary statistics on height using a procedure similar to that described for the ENIGMA data (*r*_*g*_ = 0.39 between height in UKB and uncorrected HC summary statistics from Haworth et al.^11^.

### Genotyping, imputation and quality control

The genotype data that we used was released by the UKB in March 2018, and concerns an updated version of data released earlier (July 2017). Details on the collection and processing of the genotype data are described elsewhere^17^. To summarize, the UKB genotyped in total 489,212 individuals on two custom made SNP arrays (UK BiLEVE Axiom^TM^ array covering 807,411 markers (*n* = 49,950) and UKB^TM^ Axiom array covering 825,927 markers (*n* = 438,427), both by Affymetrix). The genotype arrays shared 95% of marker content. Quality control executed by the UKB team resulted in a total of 488,377 individuals and 805,426 unique markers in the released data. In the version of the data used for the current study (genotype data release of March 2018), genotypes were phased and imputed by the UKB team to a combined reference panel of the Haplotype Reference Consortium and the UK10K. Finally, imputed and quality-controlled genotype data was available for 487,422 individuals and 92,693,895 genetic variants.

Prior to analysis, imputed variants were converted to hard call using a certainty threshold of 0.9. In our own quality control procedure, we excluded variants with a low imputation score (INFO score <0.9), low minor allele frequency (MAF < 0.005) and high missingness (>0.05). Furthermore, indels that had the same chromosomal location were excluded. This resulted in a total of 9,203,453 variants used for downstream analysis.

To reduce bias of the results due to population stratification, we only included individuals from European descent in our analyses. To this end, we projected principal components (PC’s) from the 1000 Genomes reference populations onto the called genotypes available in the UKB data. Participants for whom the projected principal component score was closest (using the Mahalanobis distance) to the average score of the European 1000Genomes sample^66^ were considered to be of European descent. Participants having a Mahalanobis distance >6 S.D. were excluded from further analysis. In an additional quality control step, we excluded participants that (1) had withdrawn their consent, (2) were related to other participants according to the UKB team (i.e., subjects with most inferred relatives, third degree or closer, were removed until no related subjects were present), (3) reported a gender that did not match their genetic gender, or (4) showed sex-chromosome aneuploidy. After filtering availability of imaging data, and MRI scan quality, 17,062 individuals remained for analysis.

### GWAS of BV in UKB

The genome-wide association analysis (GWAS) of BV in the UKB data was conducted in PLINK^67,68^, using a linear regression model with additive allelic effects. In order to correct for potential subtle population stratification effects, we included 10 genetic PC’s as covariates. Genetic PC’s were computed using FlashPCA2^69^ in the QC’ed subset of unrelated European subjects, retaining only independent (*r*^*2*^ < 0.1), relatively common (MAF > 0.01) and genotyped or very high imputation quality (INFO = 1) variants (*n* = 145,432 markers). We refer to Supplementary Note 7 for a brief comparison of the UKB BV GWAS results including either the first 10 or the first 100 genetic PC’s as covariates. Additional covariates included in the analysis were: age, sex, genotype array, assessment center, and standing height (since the genetic correlation between BV and height in the UKB is *r*_*g*_ = 0.26). We also included the TDI as covariate to correct for potential confounding factors. TDI is strongly associated with individual levels of deprivation^70^ and health-related factors including self-reported health^19^, life style factors (smoking^71^, vegetable intake^72^), chronic illness^73^, and all cause mortality^20^. We believe that inclusion of TDI as a covariate is the most appropriate correction for SES and health-related factors, since this measure has a correlational pattern with a wide variety of potential confounding factors, and because of its availability in all UKB participants.

### Conditional GWAS (mtCOJO)

Multi-trait-based conditional and joint analysis using GWAS summary data (mtCOJO^55^) can be used to conduct conditional GWAS analyses. The mtCOJO method is integrated in the GCTA^74^ software, and requires summary-level GWAS data to obtain GWAS results for phenotype A that are conditioned on the genetic signal of phenotype B. In the current study, we used mtCOJO to correct the GWAS summary statistics of ICV (ENIGMA) and head circumference (HC-GWAS) for their overlap with the genetic signal of height. The height summary statistics used in mtCOJO were obtained from a GWAS that we ran on standing height in the UKB sample (*N* = 385,748). The conditioned GWAS results were subsequently included in the GWAS meta-analysis of BV described in the following section.

### GWAS meta-analysis of BV

Before carrying out the meta-analysis of BV, we performed additional filtering and prepared the summary statistics for each of the individual cohorts. First, within the ENIGMA data and the HC-GWAS data, variants that were available for *N* < 5000 were excluded. Second, in the UKB data we identified some instances where there were multiple variants on the same position, whilst having different alleles. These variants were excluded from further analysis. Lastly, we aligned the allele coding of indels in the ENIGMA data to match the coding in the UKB data. A number of indels that were not present in the UKB data were excluded from analysis, since we used the UKB data as reference data for downstream analyses. The summary statistics from the UKB, ENIGMA, and the HC-GWAS consortium showed strong genetic correlations (*r*_*g*_ ranging between 0.75 and 1; see Supplementary Data 1), supporting our choice for a meta-analytic approach.

Using a sample-size weighted *z*-score method in METAL^26^ we combined the GWAS on BV in the UKB data (*N* = 17,062), the GWAS on ICV (conditioned on height) in the ENIGMA results (*N* = 11,373), and the GWAS on HC (conditioned on height) in the HC-GWAS results (*N* = 18,881), resulting in a total sample size of *N* = 47,316 (see Supplementary Fig. 1).

### Intelligence - GWAS summary statistics

We used recently published GWAS meta-analysis summary statistics of intelligence^53^ to study the genetic overlap with BV. Data collection procedures and methods are described in detail elsewhere^53^. In comparing the results of BV and intelligence, we updated some of the downstream analyses for intelligence (e.g., we used FUMA version 1.3.5 instead of 1.3.0, we explored a slightly different collection of gene sets, and used GTEx data v7 instead of v6.1). Therefore, gene-based findings for intelligence presented in the current study may deviate slightly from those presented in the original study^53^ (Supplementary Data 6). We applied Bonferroni correction for multiple testing to the meta-analytic variants *P*-values to identify to intelligence associated genetic variants.

### Genomic risk loci and functional annotation

We used FUMA^27^ (v1.3.5) for the functional annotation of the BV meta-analysis results. FUMA is an online platform that takes GWAS summary statistics as input, and subsequently annotates, prioritizes, and visualizes the results.

Prior to defining genomic risk loci, FUMA identifies variants that are GWS (5 × 10^−8^), and independent (*r*^*2*^ < 0.6) as independent significant variants. In the next step, independent significant variants that are independent from each other at *r*^*2*^ < 0.1 are denoted lead variants. Genotypes from the UKB were used as reference data to infer LD. Finally, FUMA characterizes genomic risk loci by merging LD blocks that are located close to each other (<250 kb apart). Thus, it is possible that one genomic risk locus contains multiple independent significant variants or lead variants.

In order to obtain information on the functional consequences of variants on genes, FUMA performs ANNOVAR^75^ gene-based annotation using Ensembl genes (build 85) for all variants in LD (*r*^*2*^ > 0.6) with one of the independent significant variants and having an association *P*-value lower than 1 × 10^−5^. In addition, Combined Annotation Dependent Depletion (CADD) scores^76^, Regulome DB scores^77^, and 15-core chromatin state^78,79^ are annotated to variants by matching chromosome position, reference, and alternative alleles. CADD scores can be used to prioritize genetic variants that are likely to be pathogenic and/or deleterious (CADD scores >12.37 suggest a variant is deleterious). The score is a single measure combining various annotations, and has been shown to correlate with pathogenicity, disease severity, and experimentally measured regulatory effects and complex trait associations^76^. RegulomeDB scores^77^ characterize variants by their likelihood to have a regulatory function (with lower scores indicating higher probability of regulatory function). Scores range from 7, meaning that there is no evidence of the variant having a regulatory function, to 1a, meaning that a variant is likely to affect binding and is linked to the expression of a gene target^77^. Chromatin state was predicted by ChromHMM^79^ for 127 cell types, using 15 states to classify and describe variants.

### Gene mapping

Using FUMA^27^, all variants in genomic risk loci that were GWS (*P* < 5 × 10^−8^) or were in LD (*r*^*2*^ > 0.6) with one of the independent significant variants, were mapped to genes. Variants could be annotated to a gene by either of three strategies. First, positional mapping maps variants to protein-coding genes based on physical proximity (i.e., within 10 kb window). Second, eQTL mapping maps variants to genes whose expression is associated with allelic variation at the variants level. Information on eQTLs was derived from three publicly available data repositories; GTEx^80^ (v7), the Blood eQTL browser^81^, and the BIOS QTL browser^82^. This strategy maps variants to genes up to 1 Mb apart (*cis*-eQTLs). We applied a false discovery rate (FDR) of ≤0.05 to limit the results to significant variant gene pairs. Third, variants were mapped to genes based on significant chromatin interactions between promoter regions of genes (250 bp up- and 500 bp downstream of the TSS) and a genomic region in a risk locus. In contrast to eQTL mapping, and in the absence of a distance boundary, chromatin interaction mapping may involve long-range interactions. The resolution of chromatin interactions was defined as 40 kb, and hence, interaction regions may comprise multiple genes. In order to prioritize genes implicated by chromatin interaction mapping, information on predicted enhancers and promoters in 111 tissue/cell types from the Roadmap Epigenomics Project^78^ was integrated. We used FUMA to filter on chromatin interactions for which one interaction region overlapped with predicted enhancers, and the other with predicted promoters 250 bp up- and 500 bp downstream of the TSS site of a gene. At the time of writing, FUMA contained Hi-C data of 14 tissue types from the study of Schmitt et al.^83^. An FDR of 1 × 10^−5^ was used to define significant interactions.

### Gene-based analysis

Genome-wide gene-based analysis (GWGAS) has the potential to identify genes associated to a trait of interest despite the genetic signal of individual variants in or nearby the gene not reaching genome-wide significance in variant-based analyses. Using the *P*-values from the variant-based analysis as input, GWGAS tests the joint signal of all variants in a gene with the phenotype, while accounting for LD between those variants. In addition to gene-mapping in FUMA, we therefore also conducted gene-based analysis in MAGMA^30^ to assess the joint effect of all variants within all 19,427 protein-coding genes included in the NCBI 37.3 database. MAGMA requires as input the *P*-values derived from variant-based analyses, in this case, the BV meta-analysis results. All variants in our BV meta-analysis were annotated to genes, resulting in 18,168 genes that contained at least one variant in the BV meta-analysis. Besides variants located within a gene, we also included variants lying within 2 kb before and 1 kb after the TSS of the gene. We used Entrez ID as the primary gene ID. MAGMA’s gene-based analysis uses a multiple linear PC regression, where an *F*-test is used to compute the gene *P*-value. The model takes linkage disequilibrium between variants into account. Genes were considered to be genome-wide significantly associated if the *P*-value survived a Bonferroni correction for multiple testing (0.05/number of genes tested: *P* < 2.75 × 10^−6^).

### Functional gene set analysis

Gene set analysis was performed using MAGMA^30^, testing 12,191 predefined gene sets in an exploratory fashion. Selected gene sets included canonical pathways (*n* = 2199) and GO gene sets (*n* = 9996). All gene sets were obtained from the Molecular Signatures Database (MSigDB, version 7.0). For all gene set analyses, competitive, rather than self-contained, *P*-values are reported. Competitive gene set analysis tests whether the joint association of genes in a gene set with the phenotype of interest is stronger than that of a randomly selected set of genes of similar size. This approach provides stronger evidence for association of the gene set compared to a self-contained test, where the joint association of genes in a gene set with the phenotype is tested against the null hypothesis of no effect.

### Gene-expression analysis

To assess whether genes associated to our traits of interest are disproportionately expressed in certain tissue- and cell types, we applied MAGMA’s gene-expression analysis to investigate associations with several gene expression profiles. First, we tested tissue gene-expression in 53 different tissue types obtained from the GTEx portal (v.7), which include gene-expression data from 13 brain tissue types^39^. Secondly, we tested gene-expression in 565 distinct adult mouse brain cell types from Dropviz^40^. These data were collected through the use of the Drop-seq technique^84^ by assessing RNA expression in 690,000 individual cells from nine brain regions of the adult mice brain, which were subsequently grouped to 565 transcriptionally distinct groups of cell types.

Gene set and gene-expression analyses were Bonferroni corrected for the total number of gene sets, tissue types and single-cell types tested in MAGMA (*P* < 3.90 × 10^−6^ (=0.05/(12,191 + 53 + 565)).

### Conditional gene set analyses

In order to gain more insight in the genetic pathways associated to BV and intelligence, we performed conditional gene set analyses using MAGMA^30^. Conditional analyses are conducted to identify MsigDB gene sets that represent independent associations (i.e., in a regression-based framework, we assessed the association between our trait of interest (e.g., BV) and a gene set, conditional on another trait (e.g., intelligence)). Specifically, we determined which gene set associations remain for BV when we condition on intelligence, and vice versa. This approach provides information on whether gene sets are uniquely associated to BV or intelligence, or rather, shared between both traits. For example, if the *P*-value of association between a gene set and BV increases when conditioning on intelligence (i.e., less significant), then this suggests that the gene set association is likely shared between both traits, while if the *P*-value between a gene set and BV is unaffected by conditioning on intelligence, then this implies that the association is specific to BV, i.e., not shared with intelligence. Note however that even when the association between a gene set and a phenotype is largely explained by a second, overlapping, gene set, its conditional *P*-value will still be reduced compared to its marginal association *P*-value. Therefore, de Leeuw et al.^38^ advise to assess the relative degree of association remaining for each gene set. In addition, we conducted pairwise conditional gene set analysis for all 18 gene sets that were significantly associated to BV. Gene-sets that showed a significant conditional *P-*value in more than half of the conditional analyses were denoted as ‘largely independent’ gene-set associations.

### SNP-based heritability and genetic correlations

We used LDSC^21^ to estimate the proportion of phenotypic variance that can be explained by common variants, a statistic known as SNP-based heritability, *h*^*2*^_SNP_. We used precomputed LD scores that were calculated using 1000 Genomes European data.

Genetic correlations (*r*_*g*_) between the signal from our BV meta-analysis, intelligence^53^, and 24 psychiatric, behavioral, and lifestyle-related traits for which summary-level data were available, were also calculated using LDSC^85^. The *r*_*g*_ estimated by LDSC is an unbiased estimate, and may exceed [−1, 1] when standard errors are large, and the genetic correlation between studies is high.

Genetic correlations for which the *P*-value survived the correction for multiple testing (Bonferroni-corrected *P* < 0.002 (=0.05/25)) were considered significant.

### Partitioned heritability

In order to determine whether some functional categories of the genome contribute more than others to the SNP-heritability (*h*^2^_SNP_) of BV, we performed stratified LDSC^29^. Using this method, we calculated whether any of the 28 specific genomic categories included in the analysis was enriched for variants that contribute to *h*^2^_SNP_. Enrichment here is defined as the proportion of *h*^2^_SNP_ in a given category divided by the proportion of variants in that category (e.g., if enrichment in intronic regions is 4.75, this indicates that this functional category has a 4.75-fold higher contribution to *h*^2^_SNP_ relative to the proportion of variants annotated to that category).

### MR analysis

MR analysis was performed using generalized summary-data-based Mendelian randomization (GSMR^55^). The main goal was to examine whether the genetic correlation between BV and intelligence (*r*_*g*_ = 0.24) might be explained by directional effects. Analyses were conducted using forward and reverse GSMR, testing for uni- and bidirectional effects between BV and intelligence. Variants that showed clear pleiotropic effects on the exposure phenotype and the outcome (i.e., instruments that are associated to both BV as well as intelligence) were excluded from analysis using the HEIDI outlier method. In addition, we excluded variants that are in LD (*r*^*2*^ > 0.1) with one another across both tests (i.e., variants that are associated to either BV or intelligence, but are in LD with a second variant that is used as an instrument for the other trait).

### Linking expression of overlapping genes to brain regions

We investigated whether genes overlapping between BV and intelligence show different brain expression patterns than non-overlapping genes, which may provide evidence of whether certain brain areas are more involved in the genetic association between BV and intelligence. To this end, we first performed gene-mapping with MAGMA and FUMA based on the summary statistics of the GWAS of each trait separately, after which we extracted the set of genes implicated by any of the gene-mapping strategies in both traits. Subsequently, we extracted the cortical gene-expression profile for each of the overlapping genes from the AHBA^61^, which describes gene-expression data per gene across distinct cortical areas. An expression profile of the set was obtained by taking the average expression across the 92 genes. We also performed clustering on the individual correlation patterns for each of the expression profiles of each of the 92 genes separately, by computing a 92 × 92 gene-to-gene correlation matrix, with the level of correlation between two genes taken as the Pearson correlation coefficient between their cortical expression profiles. This matrix was subdivided into clusters (also called modules) using Newman’s modularity algorithm^86^, maximizing for each cluster the intra-cluster cohesion and minimizing cohesion between genes of different clusters. Per cluster, a cluster-level expression profile was computed by calculating the average gene expression profile across all genes in the given cluster, resulting in clusters of genes showing distinct brain maps.

### Interaction gene set analyses

Aiming to determine whether genes associated to both BV and intelligence are over- (or under-) expressed in specific brain regions, we performed interaction analysis as implemented in MAGMA (v1.07b). This analysis tested whether the combined involvement of a gene set (here: genes related to BV) and continuous gene properties (here: gene expression) is different from their individual effects on intelligence. A positive interaction effect would suggest that, within the set of BV-related genes, the relation between expression and gene-based Z-scores for intelligence is stronger compared to other genes (i.e., all genes for which both expression data from AHBA, as well as gene-based Z-scores for intelligence were available). This would indicate that gene expression in that specific region plays a particular role in the genetic relation between BV and intelligence.

An interaction analysis was conducted separately for gene expression in each of 57 brain regions defined in the AHBA^61^. To control for potential variation in general expression levels across genes, we conditioned on the marginal effects of the BV-related gene set and average gene expression across all 57 regions, as well as for their interaction. The set of BV-related genes consisted of all genes that were significant in MAGMA’s gene-based analysis and/or mapped from variant-level results in FUMA. Interaction effects were deemed significant if their *P*-value exceeded the Bonferroni-corrected threshold of *P* < 8.77 × 10^−4^ (0.05/57).

### Reporting summary

Further information on research design is available in the Nature Research Reporting Summary linked to this article.

## Supplementary information

Supplementary Information

Peer Review File

Reporting Summary

Description of Additional Supplementary Files

Supplementary Data 1

Supplementary Data 2

Supplementary Data 3

Supplementary Data 4

Supplementary Data 5

Supplementary Data 6

Supplementary Data 7

Supplementary Data 8

Supplementary Data 9

Supplementary Data 10

Supplementary Data 11

Supplementary Data 12

Supplementary Data 13

Supplementary Data 14

Supplementary Data 15

## Data Availability

Our policy is to make genome-wide summary statistics publicly available. Summary statistics from our BV meta-analysis and the height-corrected summary statistics in UKB are available for download at the website of the Department of Complex Trait Genetics, CNCR: https://ctg.cncr.nl/software/summary_statistics. Data restrictions do not allow redistribution of the summary statistics for ICV and head circumference. To replicate our results, the uncorrected summary statistics can be requested for download from the ENIGMA and Max Planck Institute websites. The variant effect sizes can be subsequently corrected for height (summary statistics available from GWAS ATLAS; ID 3187) using the mtCOJO software. The GWAS summary statistics from the intelligence meta-analysis conducted by Savage et al.^53^ are also available from the CTG website.
