# Author Correction: Genome-wide meta-analysis of brain volume identifies genomic loci and genes shared with intelligence

**DOI:** 10.1038/s41467-022-31027-7

**Published:** 2022-06-08

**Authors:** Philip R. Jansen, Mats Nagel, Kyoko Watanabe, Yongbin Wei, Jeanne E. Savage, Christiaan A. de Leeuw, Martijn P. van den Heuvel, Sophie van der Sluis, Danielle Posthuma

**Affiliations:** 1 Department of Complex Trait Genetics, Center for Neurogenomics and Cognitive Research, Amsterdam Neuroscience, Vrije Universiteit Amsterdam, Amsterdam, The Netherlands; 2 Department of Clinical Genetics, Amsterdam UMC, Vrije Universiteit Amsterdam, Amsterdam, The Netherlands; 3 Department of Child and Adolescent Psychiatry and Psychology, Section Complex Trait Genetics, Amsterdam Neuroscience, Vrije Universiteit Medical Center, Amsterdam UMC, Amsterdam, The Netherlands

**Keywords:** Behavioural genetics, Genome-wide association studies

Correction to: *Nature Communications* 10.1038/s41467-020-19378-5, published online 5 November 2020.

In this article the grant number ERC-2018-AdG GWAS2FUNC 834057 relating to European Research Council for Danielle Posthuma was omitted. The original article has been corrected.

