# Supplementary material for: Genome-wide meta-analysis of brain volume identifies genomic loci and genes shared with intelligence
Source: Nat Commun. 2020 Nov 5;11:5606. doi: 10.1038/s41467-020-19378-5 (PMC7644755; doi:10.1038/s41467-020-19378-5)
Supplement: Supplementary file 1 — Supplementary Information [file 41467_2020_19378_MOESM1_ESM.pdf]

Supplementary Information for

Genome-wide meta-analysis of brain volume identifies genomic loci and genes shared with intelligence

Jansen et al.

Supplementary Note 1 - GWAS of brain volume in UK Biobank

After running the GWAS of brain volume (BV) in the UK Biobank data, we inspected the lambda inflation factor as well as the LD Score regression (LDSC) intercept, to check for inflation in the test statistics that is possibly due to insufficient correction for population stratification. A lambda >1 suggest an inflation of the genetic effects which can be due to both spurious and genuine effects, and is likely to increase with sample size[1]. An LDSC intercept >1 suggests that there is spurious association, and an intercept <1.10 is generally considered to suggest that the inflation of the signal is mostly due to genuine association effects. The genomic inflation factor λ_{GC} computed BV GWAS on the UKB data using LDSC, was 1.118 (**Supplementary Data 1**), suggesting minor inflation in the median test statistic compared to the expected distribution. The LDSC intercept of 1.02, however, suggested that the observed inflation in genetic signal is mostly due to polygenicity and unlikely to be driven by population stratification[1]. Specifically, the LDSC ratio of 0.14 (obtained by dividing the LDSC intercept -1 by the mean χ^2-1) indicated that as much as 86.0% of the observed inflation could be ascribed to true polygenicity and large sample size. The UKB GWAS of BV identified 3,610 genome-wide significant (GWS) variants ($P<5\times10^{-8}$) (**Supplementary Figure 3a**). FUMA then extracted 9 independent lead variants, which were mapped to 9 independent genomic loci harboring 132 genes.

Supplementary Note 2 - Using head circumference as proxy phenotype for brain volume

In contrast to the UKB and ENIGMA data, the summary statistics obtained from the head circumference (HC) GWAS are based on genetic analysis of an indirect measure of brain volume (BV), i.e., HC in children and adults. Although literature on the relation between HC and BV is fairly scarce (and most existing literature focuses on individuals with autism), we here briefly discuss research corroborating the decision to use HC as proxy for BV. An early study[2], using autopsy records comprising HC as well as brain weight measures in the same people, demonstrated a positive relationship between the two traits at least until age 18. A 2002 study[3] examined the relationship between BV and HC from childhood through adolescence. Subjects were 76 healthy, normal males of 1.7 to 42 years old (most studies that include BV measures have

focused on the relationship between BV or HC and autism, and since autism is more common in males most subjects are males). The authors reported that HC was an excellent predictor for BV in children 6 years of age or younger (r=0.93). For the older age groups in their study, HC was somewhat less strongly predictive (17-42 years old, age not included: r=0.69) or only a significant predictor when also accounting for the subjects' age (7-16 years old, age included: r=0.67). These studies suggest that there is a positive relation between HC and BV in the healthy population. Since Haworth et al. (2019)[4], whose GWAS summary statistics we use in our meta-analysis, adjusted the HC measures for age (within males and females separately) we feel that these HC summary statistics results can serve as a very good proxy measure for BV.

Supplementary Note 3 - Results of GWAS meta-analysis of BV

The main meta-analysis in the current study combined BV GWAS results from the UK Biobank (UKB; N=17,062, ran specifically for the current analyses) with ICV GWAS results from the ENIGMA consortium (N=11,373)[5] and data on head circumference (HC) from a previous large GWAS meta-analysis (N=18,881)[4], resulting in a final meta-analysis of brain volume in N=47,316 individuals (**Supplementary Figure 1**). The meta-analysis was run in METAL[6] using a sample-size weighted *z*-score method.

In order to assess whether there was inflation in the observed test statistics of the meta-analysis compared to expected values, we inspected the Lambda GC (λ_{GC}) and the LDSC intercept of the meta-analytic results (**Supplementary Data 1**). A λ_{GC} >1 suggests an inflation of the genetic effects, which can be due to both spurious and genuine effects. The value of λ_{GC} is likely to increase with sample size[1]. An LDSC intercept >1 suggests that there is spurious association, although an intercept <1.10 is usually considered to suggest that the signal is mostly due to genuine association effects[7]. For the current meta-analysis, the λ_{GC} was 1.18, indicating that, compared to the expected distribution, the median test statistic was inflated. The LDSC intercept of 1.025, however, suggested that the observed inflation is mostly due to genuine polygenicity, rather than caused by population stratification. The BV meta-analysis identified 4,155 genome-wide significant variants, which FUMA[8] ascribed to 18 genomic loci. Lead variants across all 18 independent genomic risk loci had the same direction of effect in all cohorts in which they were available

(**Supplementary Data 3**), thereby extending our confidence in the results. One additional locus reported in FUMA was deemed unreliable, as it appeared to be driven by a single nucleotide polymorphism (SNP) (12:12871099:G_T or rs2066827; see **Supplementary Figure 4**). The SNP of interest had a MAF of 0.23 in the UKB (compared to ~0.36 in the 1000Genomes and TOPMED data), and was not in LD with other variants in the 1000Genomes data.

Supplementary Note 4 - Functional annotation of GWAS meta-analysis of BV

To extract additional biological information from the GWAS results, we ran functional annotation analyses using the FUMA[8] annotation platform. Annotation was performed on all variants that were considered to be a *candidate variant* given a) high LD with one of the independent significant variant (r^2>0.6) and b) a low *P*-value ($P<1\times10^{-5}$), which resulted in 4,683 *candidate variants* (**Supplementary Data 4**). We observed that most of these variants were located within intergenic regions (n=751, 16.0%) or intronic regions (n=2.444, 52.2%), and a small minority of variants in exonic regions (n=62, 1.3%; **Supplementary Figure 5**).

Of the 62 variants annotated to exonic regions, 27 SNPs were associated with non-synonymous changes in the proteins of the gene (ExNS; **Supplementary Data 5**). These ExNS SNPs were located in exons of 13 genes, with several genes including multiple ExNS SNPs, e.g., the *SPPL2C* gene contained 8 ExNS SNPs, all located in exon 1 of this gene. This gene codes for the signal peptide peptidase-like 2C, an intramembrane protease (IMP) that has been shown to play a role in the degradation of signaling peptides in the brain[9], of which its exact biological substrate remains largely unclear[10], but which has recently been suggested to protease SNARE proteins[11], involved in vesicle transport and neurotransmitter release[12]. In addition, the *MAPT* gene contained 6 ExNS SNPs, which were located in exon 1 (a stopgain), 6 and 8. The *MAPT* gene product is the Microtubule Associated Protein Tau protein, a protein that regulates the assembly of microtubules[13], and which has been linked to a wide variety of neurodegenerative disorders including Alzheimer's disease[14], frontotemporal dementia[15] and Parkinson's disease[16].

Supplementary Note 5 - Gene mapping results of GWAS meta-analysis of BV

To find genes for BV, we used three strategies to map variants to genes in FUMA[8], as well as genome-wide gene-based association analysis (GWGAS) conducted in MAGMA[17] (**Methods**; **Supplementary Figure 6**). Summary statistics of the meta-analysis of BV (UKB, ENIGMA, HC-GWAS) were used as input for FUMA. FUMA gene-mapping mapped the 18 genomic loci to 119 genes through positional mapping (locus near to, or within a gene), 207 genes through eQTL mapping (locus associated with the expression of a gene), and 192 genes through chromatin-chromatin interactions (locus that physically interacts with a close or distant gene through the 3D structure of the genome), leading to a set of 321 unique genes (**Fig 2a**; **Supplementary Data 6)**. Of these genes, 156 genes (49.1%) were mapped by more than one gene-mapping method and 40 genes (12.6%) by all three mapping methods. Of all 321 genes mapped by FUMA, 59 genes had a pLI > 0.90, suggesting extreme intolerance to loss of function (LoF) mutations (**Supplementary Data 6**). In addition, genome-wide gene-based analysis (GWGAS) was carried out in MAGMA[17] . GWGAS identified 69 significant genes (**Fig. 2b**; **Supplementary Data 7**), of which a large part (11 genes, 15.9%) were mapped on chromosome 17. Of the 69 significant genes, 44 genes (63.8%) were also observed in FUMA, and 16 genes (23.2%) were implicated through all four mapping strategies (GWGAS, positional mapping, eQTL mapping and chromatin-chromatin interaction mapping; *FRZB, FOXO3, WBP1L, INA, PRR13, MAP3K12, RAB5B, SUOX, RPS26, ERBB3, HMGA2, PITPNM2, C12orf65, PLEKHM1, MAPT, KANSL1*). Of the 16 genes that were observed in all gene-mapping methods, several genes are known to be important in regulatory functions of cell-signaling and cell neuronal development. For instance, the *FOXO3* gene is part of the forkhead gene-family that code for transcription factors involved in cell survival. Transcription factors coded by *FOXO3* are phosphorylated by *AKT1*[18] and induce cell death through triggering of apoptosis. Overexpression of this gene has been shown to lead to cell growth decline in cell lines[19]. *ERBB3* (also known as *HER3*: human epidermal growth factor receptor 3) is part of the epidermal growth factor receptor tyrosine kinases and is a receptor for the heregulin protein[20]. Activation of the ERBB3 receptor leads to activation of the PI3K/ATK-signaling pathway which modifies cell differentiation and proliferation[21].

Supplementary Note 6 - Gene-set results of GWAS meta-analysis of BV

We performed gene-set analysis in MAGMA[17], using *P*-values of 18,169 genes as input from the prior GWGAS gene-based testing, and tested 12,191 gene-sets extracted from the online molecular signatures database (MsigDB, version 7.0;) repository[22] for their association with BV. Selected gene sets included expert-curated gene sets and gene ontology (GO) gene sets. We identified 18 significant gene-sets (**Supplementary Data 10**). Almost all gene-sets showed partial overlap in genes with each other (**Supplementary Figure 7**), implying that several significantly associated genes were shared between those gene-sets **(Supplementary Figure 8**). Among these genes were *ERBB3* ($n_{genesets}$=7, gene-based $P=1.54\times10^{-11}$), *FOXO3* ($n_{genesets}$=10; gene-based $P=3.27\times10^{-14}$) and *WNT3* ($n_{genesets}$=11, gene-based $P=2.05\times10^{-18}$). The *ERBB3* gene (Erb-B2 Receptor Tyrosine Kinase 3) is a receptor tyrosine kinase of the ERBB family that plays an essential role as cell surface receptor for neuregulins. Overexpression of this gene's protein has been reported to be related to various types of cancer. *FOXO3,* forkhead box O3 (see also **Supplementary Note 5**), is a transcription factor and a key factor in a wide array of cellular functions, including cell proliferation, differentiation and survival[23]. *WNT3* (Wnt family member 3) is part of a gene family that encodes secreted signaling proteins. Gene expression studies indicate an important role for this gene in some cases of human breast, rectal, lung, and gastric cancer through activation of the WNT-beta-catenin-TCF signaling pathway. The gene has also been linked to Tetra-Amelia Syndrome. These results suggest that similar regulatory biological pathways are involved in normal cell division during brain development as well as in aberrant cell division in the development of cancerous tumors in several types of cancer, which explains why we observed significant enrichment of genes in endometrial cancer and PI3K/AKT Signaling in Cancer gene-sets.

Since gene-sets are hierarchically structured with several gene-sets being partly nested within each other, some gene-sets may be implicated through their dependence on other gene-sets. To investigate whether the identified gene-sets were independently associated with BV, we carried out conditional gene-set analyses[24]. Conditional gene-set analysis calculates *P*-values for each gene-set conditional on the association of each of the other gene-sets. These analyses showed that 9 of the 18 gene-sets displayed largely independent signals (defined as conditional *P*-values that remained significant in more than half (i.e. more than 8) of the

conditional analyses, **Supplementary Figure 9, Supplementary Data 12**). The gene-sets with the largest number of conditional *P*-values remaining significant were REACTOME: IRS activation (n=16), REACTOME: Formation of senescence-associated heterochromatin foci SAHF (n=16), GO: Growth (n=15), suggesting that these gene-set were the most independent from the other implicated gene-sets.

Supplementary Note 7 - Comparison with GWAS in UKB using 100 genetic principal components

As per the request of an anonymous reviewer, we repeated the GWAS of BV in the UK Biobank data, using the first 100 genetic (PCs) rather than the first 10 PCs, as we decided to use in the GWAS on the UKB data that was included in our meta-analysis (**Supplementary Figure 11**). Genetic PCs were computed on the same data, using the same software and the same procedure as applied for computation of the first 10 PCs. The LD Score intercept in the 100PC-corrected GWAS (LDSC intercept = 1.020, SE= 0.007) remained practically unchanged compared to the 10PC-corrected GWAS (LDSC intercept = 1.020, SE=0.007). The genetic correlation, as computed using LD Score regression, between both GWAS was approximately 1 (r_g = 0.989, SE=0.002), alleviating concerns that 10 PCs would be insufficient to correct for subtle population stratification.

Subsequently, we conducted an additional meta-analysis of BV, using the results of the 100PC-corrected GWAS of BV in the UKB rather than our original 10PC-corrected UKB GWAS. The genetic correlation between the original meta-analysis and this second meta-analysis was 0.9979 (SE=0.0004). Given that we only observe minimal changes in the results when adding up to 100 PCs as covariates in the analyses, we conclude that most of the confounding due to population stratification was corrected for in the 10PC-corrected GWAS in UK Biobank. All the results discussed in the main text are based on the 10PC-corrected UKB GWAS.

Supplementary Figures

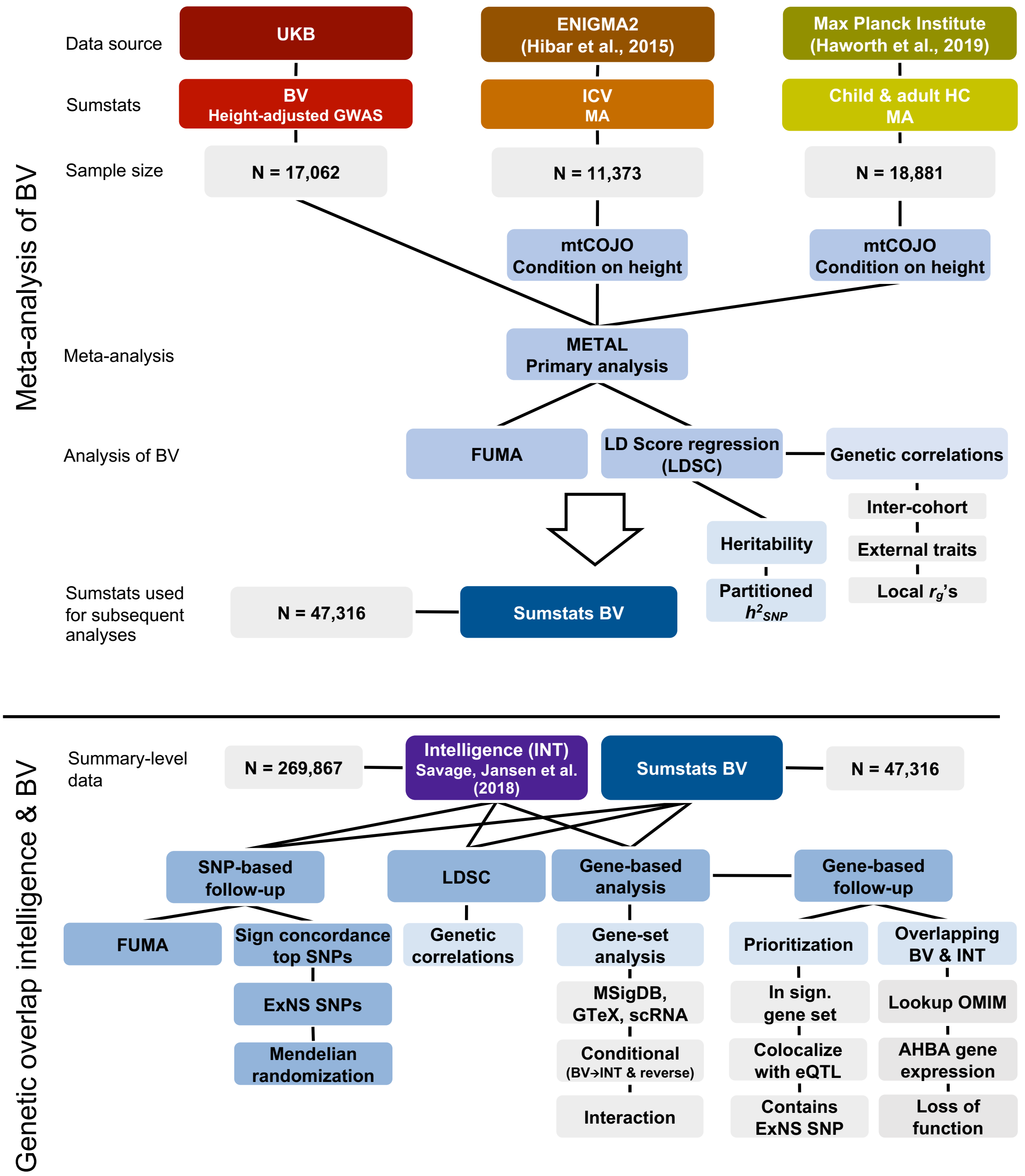

Supplementary Fig. 1. Flowchart of the GWAS meta-analyses of brain volume. Schematic representation of the analyses conducted in the current study. ICV = intracranial volume; HC = head circumference; BV = brain volume; INT = Intelligence; MA = meta-analysis.

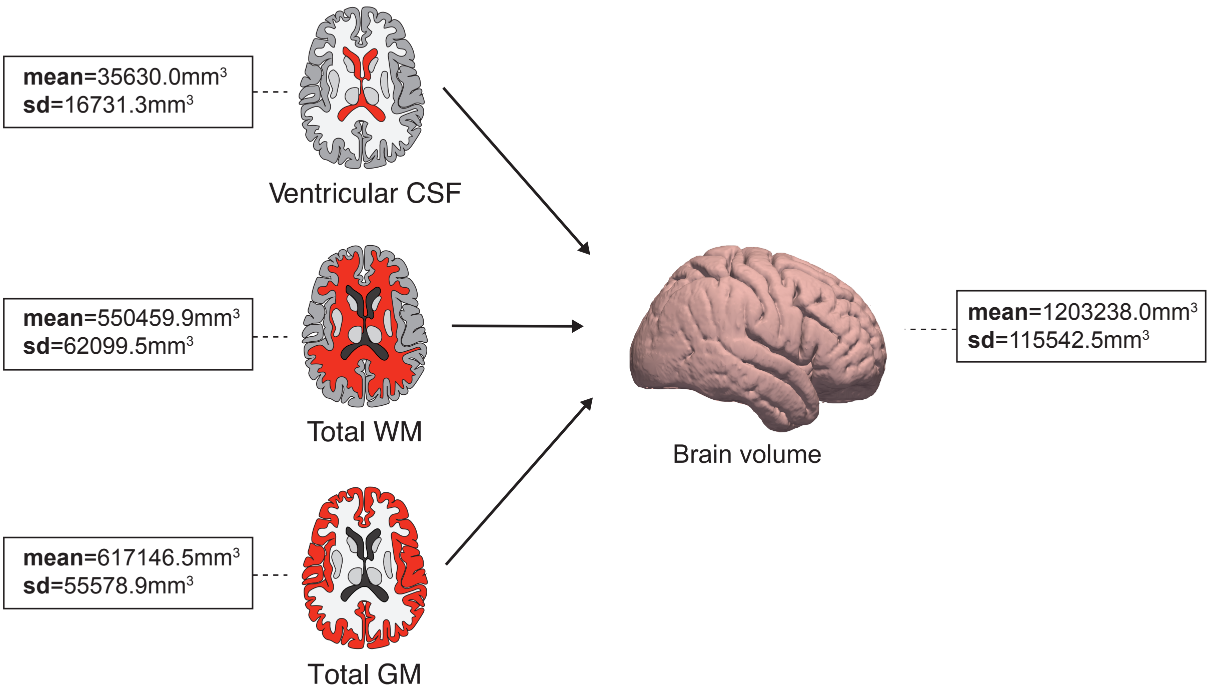

Supplementary Fig. 2. Distribution of brain structures included in the BV phenotype in the UK Biobank. Mean and standard deviation (sd) of the three brain phenotypes that were combined in the brain volume measure in the UK Biobank data (N=17,062). Brain volume means and sd's are expressed in cubic millimeters (mm^3). CSF=cerebrospinal fluid; WM=white matter; GM=gray matter.

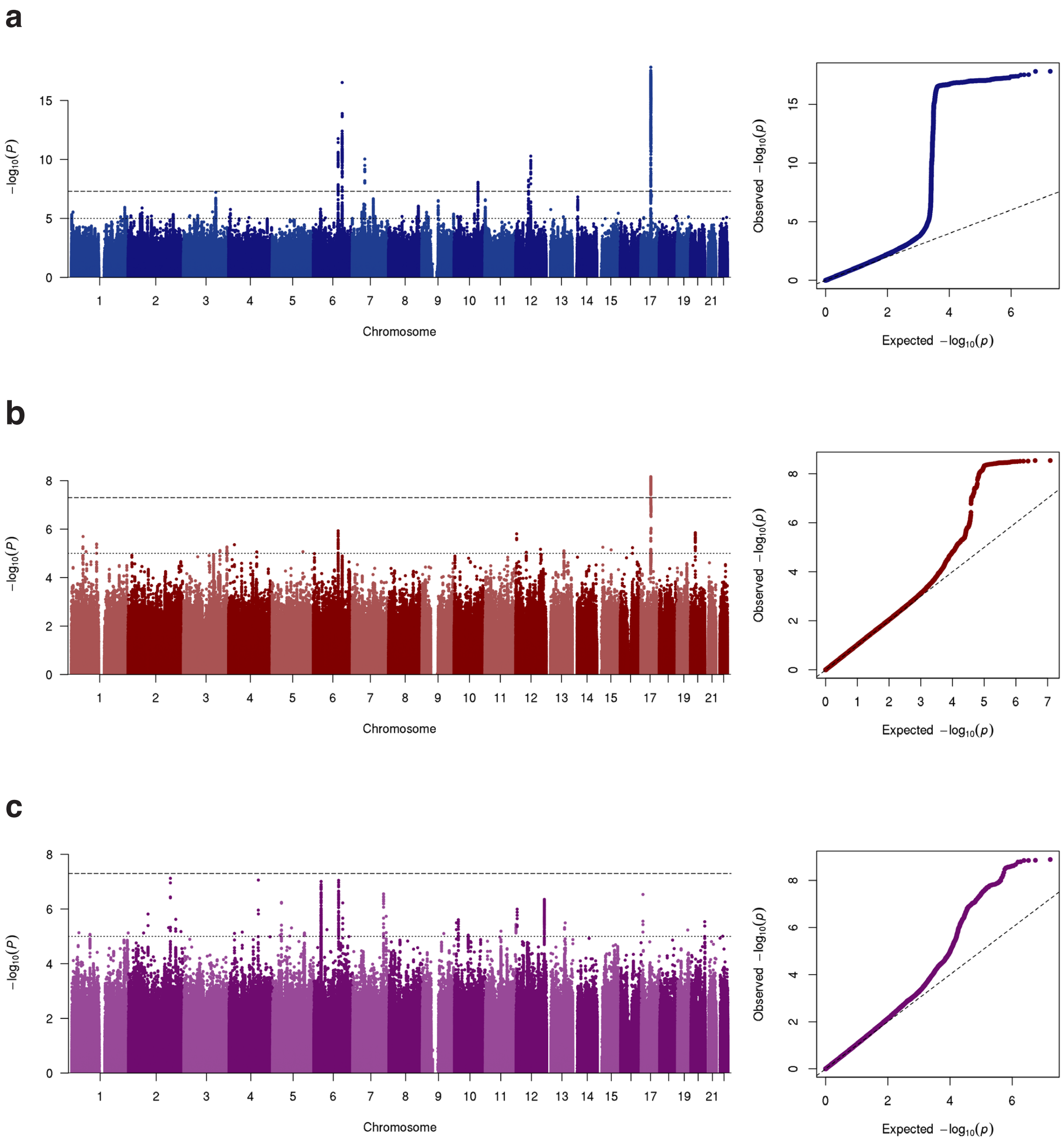

Supplementary Fig. 3. Genome-wide association analyses of brain volume included in the meta-analysis. Manhattan plot showing negative $\log_{10}$-transformed *P*-values on the *y*-axis and genomic position on the *x*-axis, and QQ-plots showing the observed versus the expected *P*-value distribution. Results are shown for **(a)** the GWAS of brain volume in UK Biobank (N=17,062 individuals), **(b)** the GWAS of intracranial volume in the ENIGMA consortium (N=11,373 individuals), and **(c)** the GWAS of head circumference (N=18,881 individuals). The data for the latter two samples was corrected for height using mtCOJO (see **Methods**). The upper horizontal dotted line corresponds to a *P*-value of $P < 5\times10^{-8}$, while the lower horizontal dotted line indicates the suggestive threshold of $P < 1\times10^{-5}$.

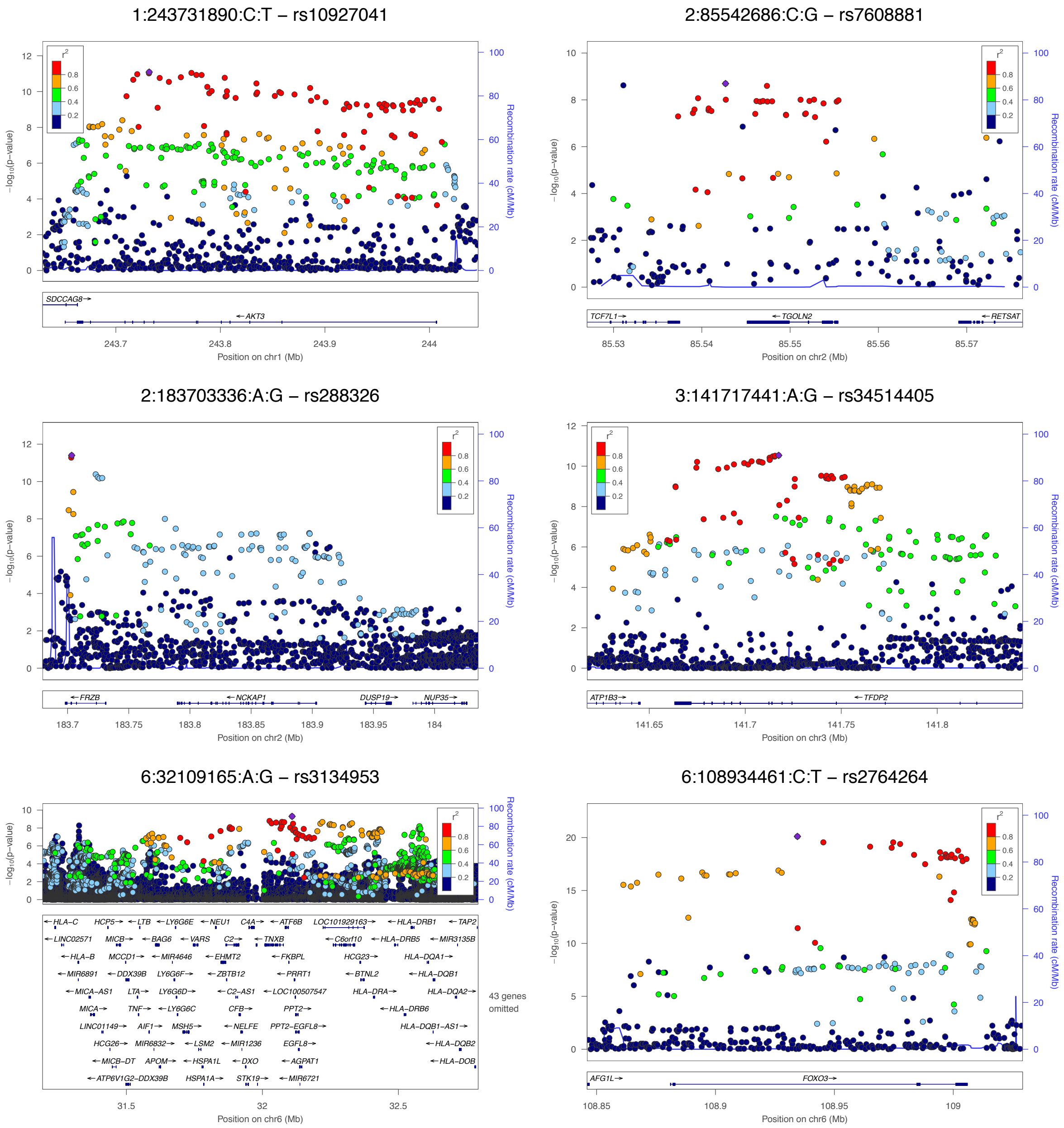
1:243731890:C:T – rs10927041
2:85542686:C:G – rs7608881
2:183703336:A:G – rs288326
3:141717441:A:G – rs34514405
6:32109165:A:G – rs3134953
6:108934461:C:T – rs2764264
−log10(p−value)
Recombination rate (cM/Mb)
r2
0.8
0.6
0.4
0.2
SDCCAG8
AKT3
Position on chr1 (Mb)
TCF7L1
TGOLN2
RETSAT
Position on chr2 (Mb)
FRZB
NCKAP1
DUSP19
NUP35
Position on chr2 (Mb)
ATP1B3
TFDP2
Position on chr3 (Mb)
HLA−C
HCP5
LTB
LY6G6E
NEU1
C4A
ATF6B
LOC101929163
HLA−DRB1
TAP2
LINC02571
MICB
BAG6
VARS
C2
TNXB
C6orf10
HLA−DRB5
MIR3135B
HLA−B
MCCD1
MIR4646
EHMT2
FKBPL
HCG23
HLA−DQA1
MIR6891
DDX39B
LY6G6F
ZBTB12
PRRT1
BTNL2
HLA−DQB1
MICA−AS1
LTA
LY6G6D
C2−AS1
LOC100507547
HLA−DRA
HLA−DQA2
MICA
TNF
LY6G6C
CFB
PPT2
HLA−DRB6
LINC01149
AIF1
MSH5
NELFE
PPT2−EGFL8
HLA−DQB1−AS1
HCG26
MIR6832
LSM2
MIR1236
EGFL8
HLA−DQB2
MICB−DT
APOM
HSPA1L
DXO
AGPAT1
HLA−DOB
ATP6V1G2−DDX39B
HSPA1A
STK19
MIR6721
43 genes omitted
Position on chr6 (Mb)
AFG1L
FOXO3
Position on chr6 (Mb)

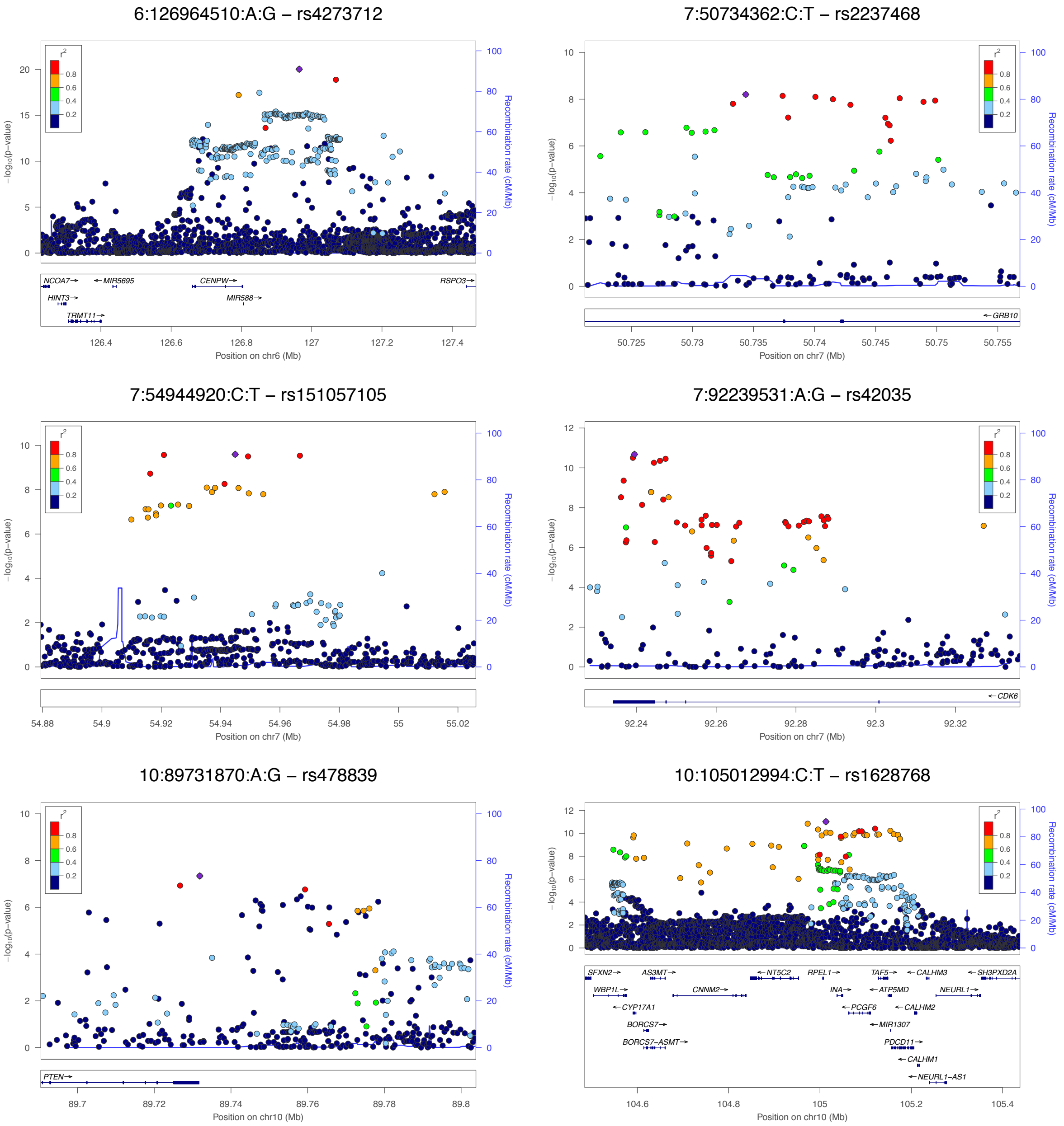

6:126964510:A:G – rs4273712
NCOA7→ ←MIR5695 CENPW→ RSPO3→
HINT3→ MIR588→
TRMT11→
Position on chr6 (Mb)
7:50734362:C:T – rs2237468
←GRB10
Position on chr7 (Mb)
7:54944920:C:T – rs151057105
Position on chr7 (Mb)
7:92239531:A:G – rs42035
←CDK6
Position on chr7 (Mb)
10:89731870:A:G – rs478839
PTEN→
Position on chr10 (Mb)
10:105012994:C:T – rs1628768
SFXN2→ AS3MT→ ←NT5C2 RPEL1→ TAF5→ ←CALHM3 ←SH3PXD2A
WBP1L→ CNNM2→ INA→ ←ATP5MD NEURL1→
←CYP17A1 ←PCGF6 ←CALHM2
BORCS7→ ←MIR1307
BORCS7–ASMT→ PDCD11→
←CALHM1
←NEURL1–AS1
Position on chr10 (Mb)
−log10(p−value)
Recombination rate (cM/Mb)

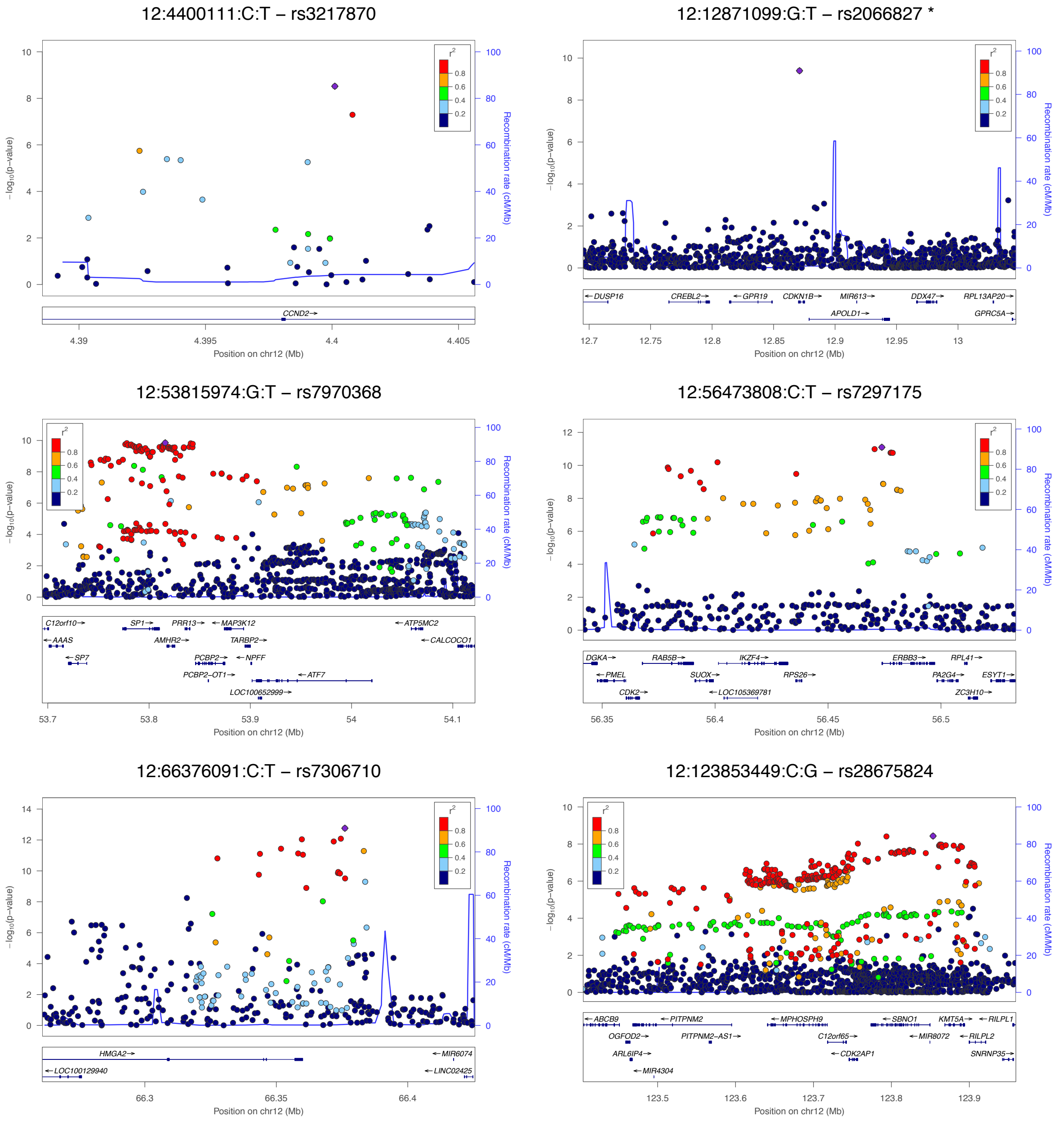
12:4400111:C:T – rs3217870
12:12871099:G:T – rs2066827 *
12:53815974:G:T – rs7970368
12:56473808:C:T – rs7297175
12:66376091:C:T – rs7306710
12:123853449:C:G – rs28675824
−log10(p−value)
Recombination rate (cM/Mb)
Position on chr12 (Mb)
r2
CCND2
DUSP16
CREBL2
GPR19
CDKN1B
MIR613
APOLD1
DDX47
RPL13AP20
GPRC5A
C12orf10
AAAS
SP7
SP1
AMHR2
PRR13
PCBP2
PCBP2−OT1
MAP3K12
TARBP2
NPFF
LOC100652999
ATF7
ATP5MC2
CALCOCO1
DGKA
PMEL
CDK2
RAB5B
SUOX
IKZF4
LOC105369781
RPS26
ERBB3
PA2G4
RPL41
ESYT1
ZC3H10
HMGA2
LOC100129940
MIR6074
LINC02425
ABCB9
OGFOD2
ARL6IP4
MIR4304
PITPNM2
PITPNM2−AS1
MPHOSPH9
C12orf65
CDK2AP1
SBNO1
MIR8072
KMT5A
RILPL2
RILPL1
SNRNP35

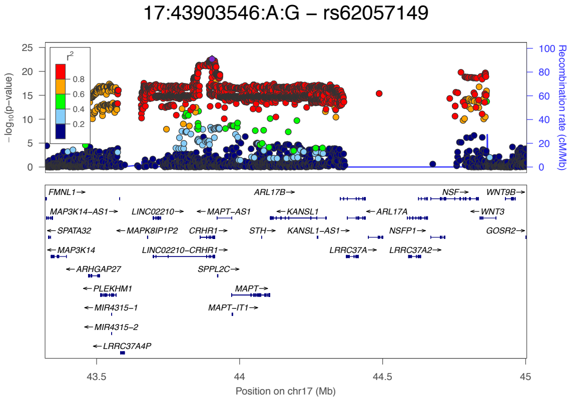

Supplementary Fig. 4. Regional association plots of the meta-analyzed variant results for BV. Regional plots were created using LocusZoom for all genomic loci identified in the GWAS meta-analysis of BV (N=47,316 individuals). The *y*-axis represents the negative $\log_{10}$-transformed variant *P*-values and the *x*-axis indicates the genomic position. Level of LD between a variant and the lead variant (purple) is indicated by the color. The low-confidence locus (12:12871099:G_T) is indicated by an asterisk.

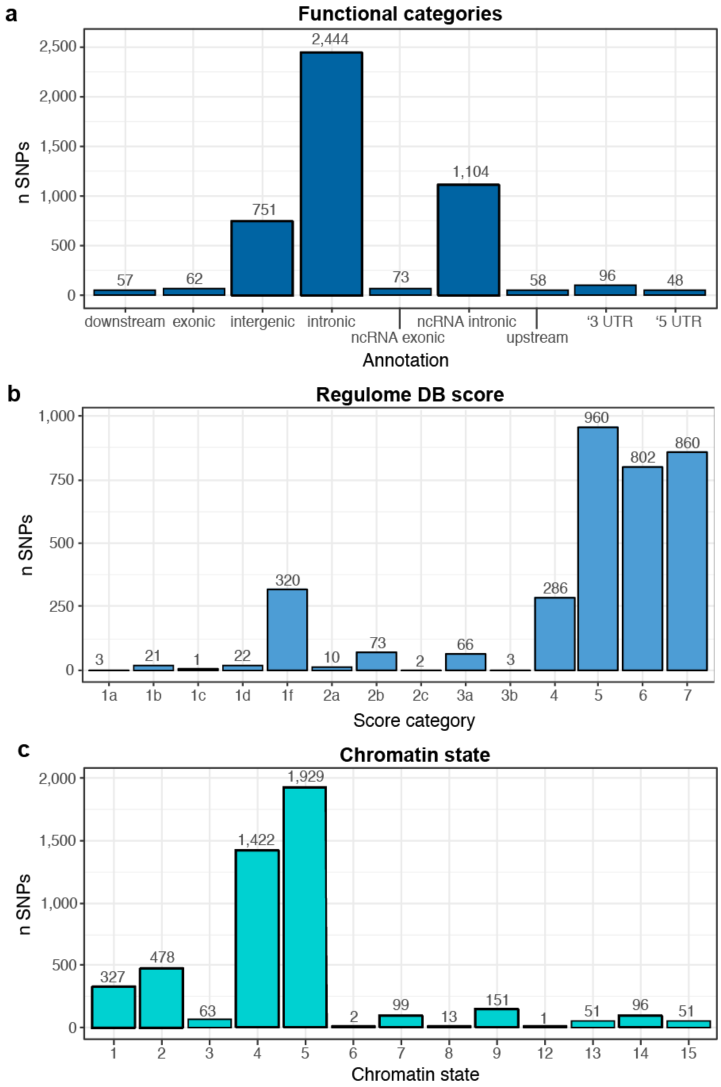

Supplementary Fig. 5. Functional annotation of the GWAS meta-analysis results of BV (N=47,316). Functional annotation of the BV meta-analysis results was carried out in FUMA on all 4,683 *candidate variants* (LD > 0.6 with an independent significant variant and *P*-value of < 1×10^{-5}). **(a)** Number of variants annotated to the functional categories. **(b)** Number of variants in each Regulome DB score category. A lower score represents a higher likelihood that the variant has a regulatory function. (**c**) Number of variants in each of 15 possible chromatin states.

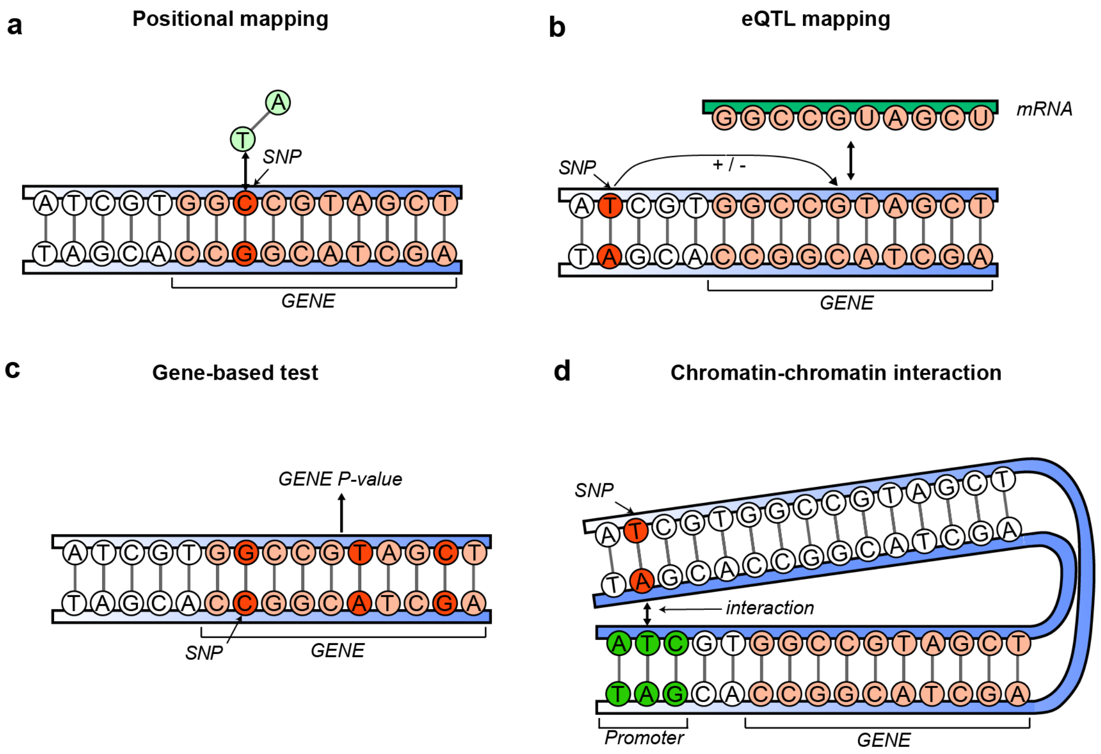

Supplementary Fig. 6. Gene-mapping methods that were used to map variants from GWAS to genes. Schematic representation of gene-mapping efforts used in the GWAS follow-up analyses: **(a)** positional gene-mapping based on position of genome-wide significant (GWS) variants within the physical boundaries (or within proximity, i.e., within 10 kb window) of a gene, **(b)** gene-mapping through eQTL, where a GWS variant is known to influence the expression levels of a gene, **(c)** gene-based association testing (MAGMA), where variant association *P*-values within a gene are combined into a gene-based *P*-value, and **(d)** gene-mapping through chromatin-chromatin interaction, where GWS variants physically interact with genes through the 3D structure of the genome.

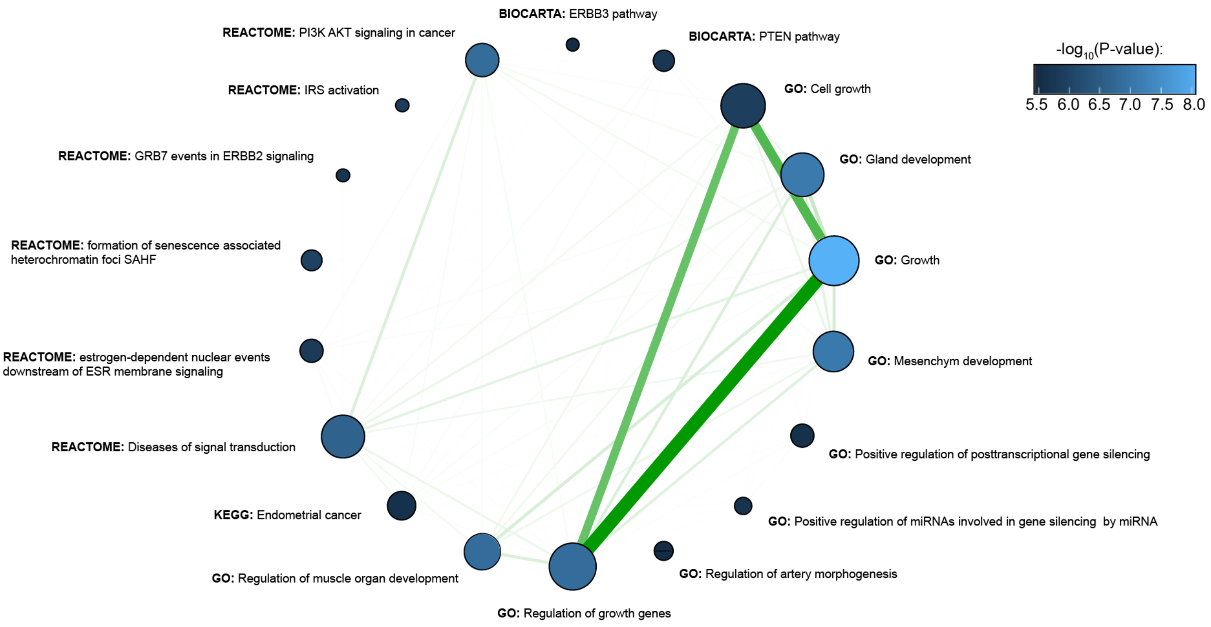

Supplementary Fig. 7. Significant gene sets in the GWAS meta-analysis of brain volume. Gene-set analysis was performed in MAGMA using gene-based *P*-values as input. Gene sets are shown that passed the stringent Bonferroni correction for multiple testing ($P < 0.05/12{,}191 = 4.10\times10^{-6}$). The number of genes in the gene set are represented by the size of the circle, the -$\log_{10}$-transformed *P*-value of the gene set by the color of the circle, and the number of genes that overlap between gene sets by the strength of the connections.

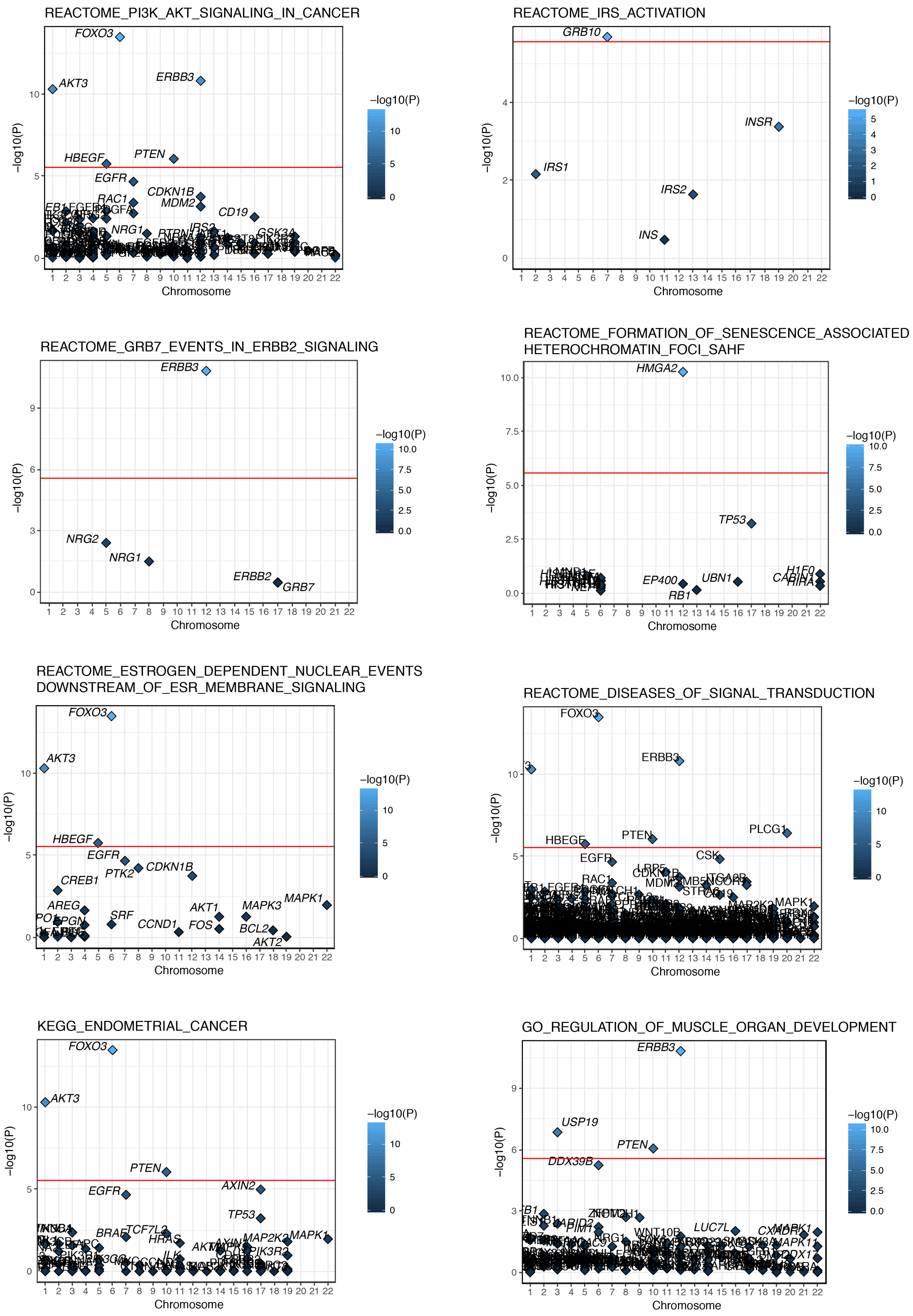
REACTOME_PI3K_AKT_SIGNALING_IN_CANCER
FOXO3
AKT3
ERBB3
HBEGF
PTEN
EGFR
CDKN1B
MDM2
CD19
REACTOME_IRS_ACTIVATION
GRB10
INSR
IRS1
IRS2
INS
REACTOME_GRB7_EVENTS_IN_ERBB2_SIGNALING
ERBB3
NRG2
NRG1
ERBB2
GRB7
REACTOME_FORMATION_OF_SENESCENCE_ASSOCIATED
HETEROCHROMATIN_FOCI_SAHF
HMGA2
TP53
EP400
UBN1
RB1
REACTOME_ESTROGEN_DEPENDENT_NUCLEAR_EVENTS
DOWNSTREAM_OF_ESR_MEMBRANE_SIGNALING
FOXO3
AKT3
HBEGF
EGFR
CDKN1B
PTK2
CREB1
AREG
SRF
AKT1
MAPK3
MAPK1
CCND1
FOS
BCL2
AKT2
REACTOME_DISEASES_OF_SIGNAL_TRANSDUCTION
FOXO3
ERBB3
PLCG1
HBEGF
PTEN
EGFR
CSK
KEGG_ENDOMETRIAL_CANCER
FOXO3
AKT3
PTEN
EGFR
AXIN2
TP53
BRAF
TCF7L2
HRAS
MAPK1
GO_REGULATION_OF_MUSCLE_ORGAN_DEVELOPMENT
ERBB3
USP19
PTEN
DDX39B
LUC7L
−log10(P)
Chromosome

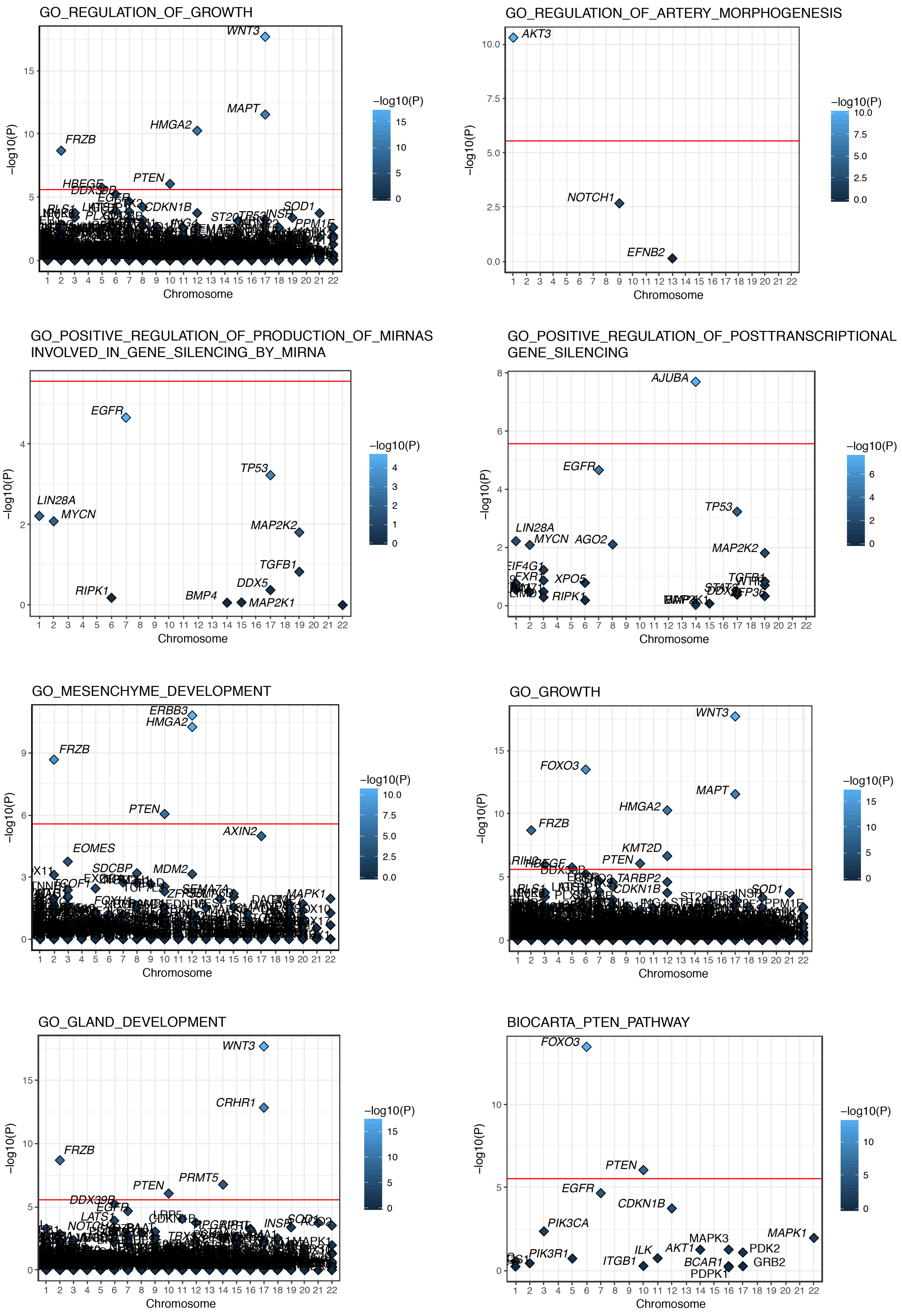

GO_REGULATION_OF_GROWTH
WNT3
MAPT
HMGA2
FRZB
PTEN
CDKN1B
SOD1
−log10(P)
Chromosome
GO_REGULATION_OF_ARTERY_MORPHOGENESIS
AKT3
NOTCH1
EFNB2
−log10(P)
Chromosome
GO_POSITIVE_REGULATION_OF_PRODUCTION_OF_MIRNAS INVOLVED_IN_GENE_SILENCING_BY_MIRNA
EGFR
TP53
LIN28A
MYCN
MAP2K2
TGFB1
DDX5
RIPK1
BMP4
MAP2K1
−log10(P)
Chromosome
GO_POSITIVE_REGULATION_OF_POSTTRANSCRIPTIONAL GENE_SILENCING
AJUBA
EGFR
TP53
LIN28A
MYCN
AGO2
MAP2K2
EIF4G1
XPO5
RIPK1
TGFB1
−log10(P)
Chromosome
GO_MESENCHYME_DEVELOPMENT
ERBB3
HMGA2
FRZB
PTEN
AXIN2
EOMES
SDCBP
MDM2
MAPK1
−log10(P)
Chromosome
GO_GROWTH
WNT3
FOXO3
MAPT
HMGA2
FRZB
KMT2D
PTEN
TARBP2
CDKN1B
SOD1
−log10(P)
Chromosome
GO_GLAND_DEVELOPMENT
WNT3
CRHR1
FRZB
PRMT5
PTEN
DDX39B
EGFR
LATS1
−log10(P)
Chromosome
BIOCARTA_PTEN_PATHWAY
FOXO3
PTEN
EGFR
CDKN1B
PIK3CA
MAPK3
MAPK1
PIK3R1
ILK
AKT1
ITGB1
BCAR1
PDK2
GRB2
PDPK1
−log10(P)
Chromosome

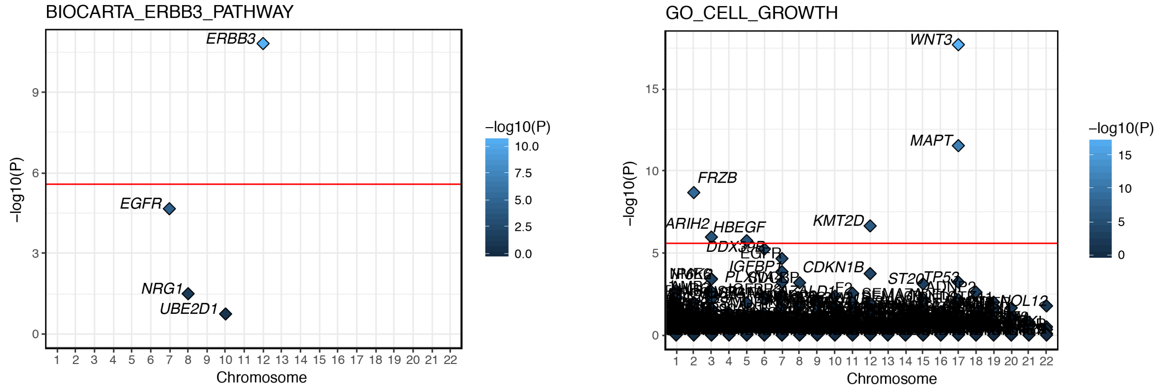

Supplementary Fig. 8. Gene associations within each of the significant gene set. Gene-based *P*-values are shown for all genes that are part of one of the 18 gene sets significantly associated with BV in MAGMA (gene-sets $P <$ 4.10×10^{-6}; i.e. $P< 0.05/12{,}191$). The chromosomal location of each gene within the gene set is shown on the x-axis, whereas the $-\log_{10}$-transformed *P*-value of the gene in the gene-based test in MAGMA is shown on the y-axis and is represented by the dot color. Genes are annotated by gene name. The horizontal red line corresponds to the Bonferroni corrected significance threshold of $P < 0.05/18{,}169$ in the gene-based test.

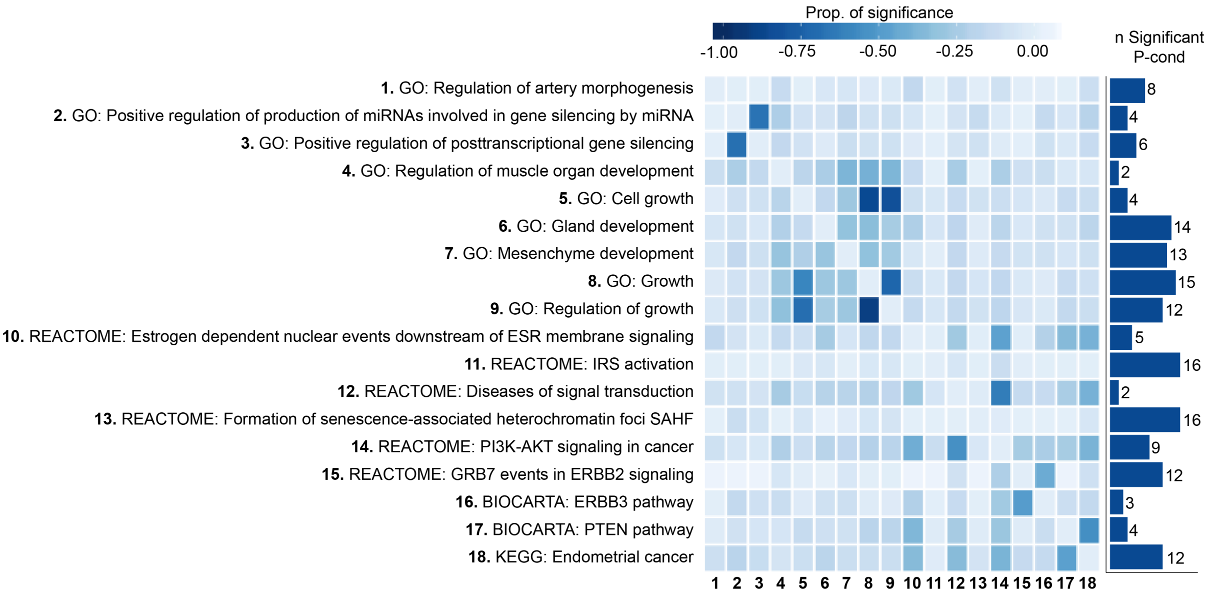

Supplementary Fig. 9. Conditional gene-set analysis. Heatmap of the pairwise conditional gene-set analyses of the 18 gene-sets that were significantly associated to BV. Off-diagonal entries represents the scaled difference between negative $\log_{10}$-transformed *P*-values of conditional minus marginal gene-set association *P*-values (Proportion of significance = ($-\log_{10}(P\_cond)$ minus $-\log_{10}(P\_marg)$) / $-\log_{10}(P\_marg)$; with more negative values indicating that the marginal P-value is lower than the conditional *P*-value, suggesting decrease of the significance of the gene set after conditioning. Gene-sets on the y-axis were conditioned on the gene-set on the x-axis. The bar plot on the right shows the number of conditional *P*-values that remained significant for each gene-set conditioning on each of the other significant gene-sets. All (conditional) gene-set analyses were performed in MAGMA.

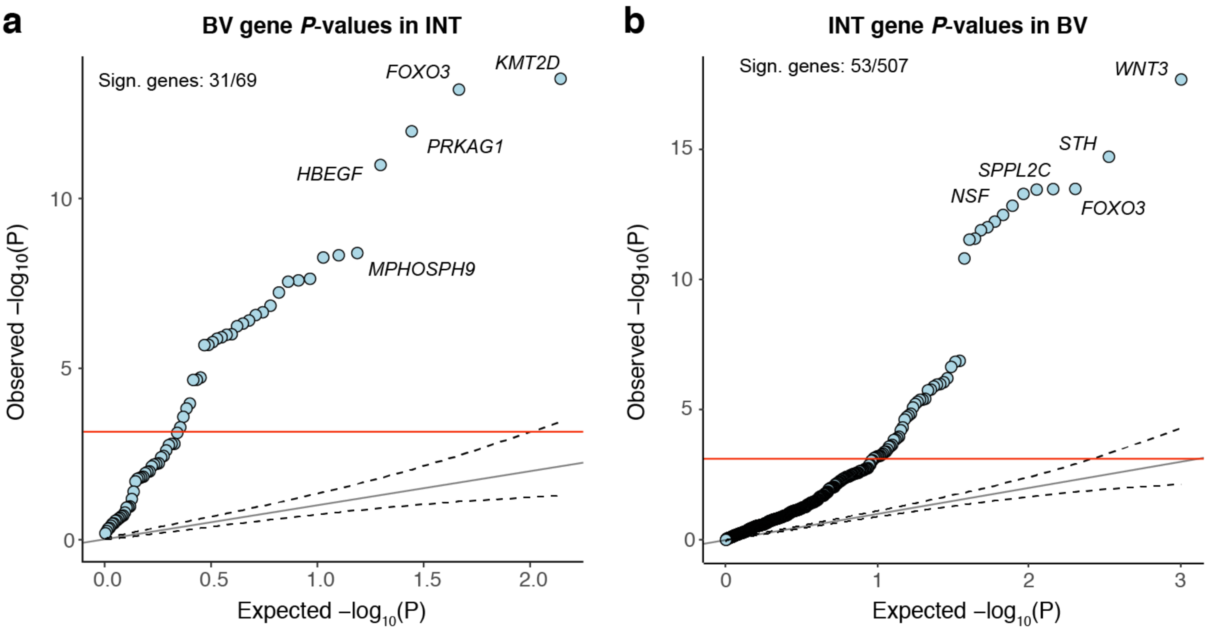

Supplementary Fig. 10. Gene *P*-values of significant brain volume genes in intelligence, and vice versa. The *x*-axis shows the expected gene *P*-values under the H_0 of no genetic overlap and the *y*-axis the observed gene *P*-value. For all significant genes in the gene-based analysis of BV we looked up the *P*-value of association for INT, and vice versa. Panel **(a)** shows the gene *P*-values from the meta-analysis of INT for the 69 genes that were identified for BV. Panel **(b)** shows the gene *P*-values from the meta-analysis of BV for the 507 genes that were identified for INT. The dashed lines represent the upper and lower limit of the 95% confidence interval around the observed-*P*-value-equals-expected-*P*-value line. The red line indicates the Bonferroni-corrected threshold (**a**: $P<0.05/69$ genes = 7.24×10^{-4}; **b**: $P<0.05/507=9.86\times10^{-5}$). The number in the upper left corner indicates the number of BV-related genes that were also significant in INT, and vice versa (e.g., in panel a; of the 69 genes identified in the gene-based analysis of BV, 31 were also significant for INT).

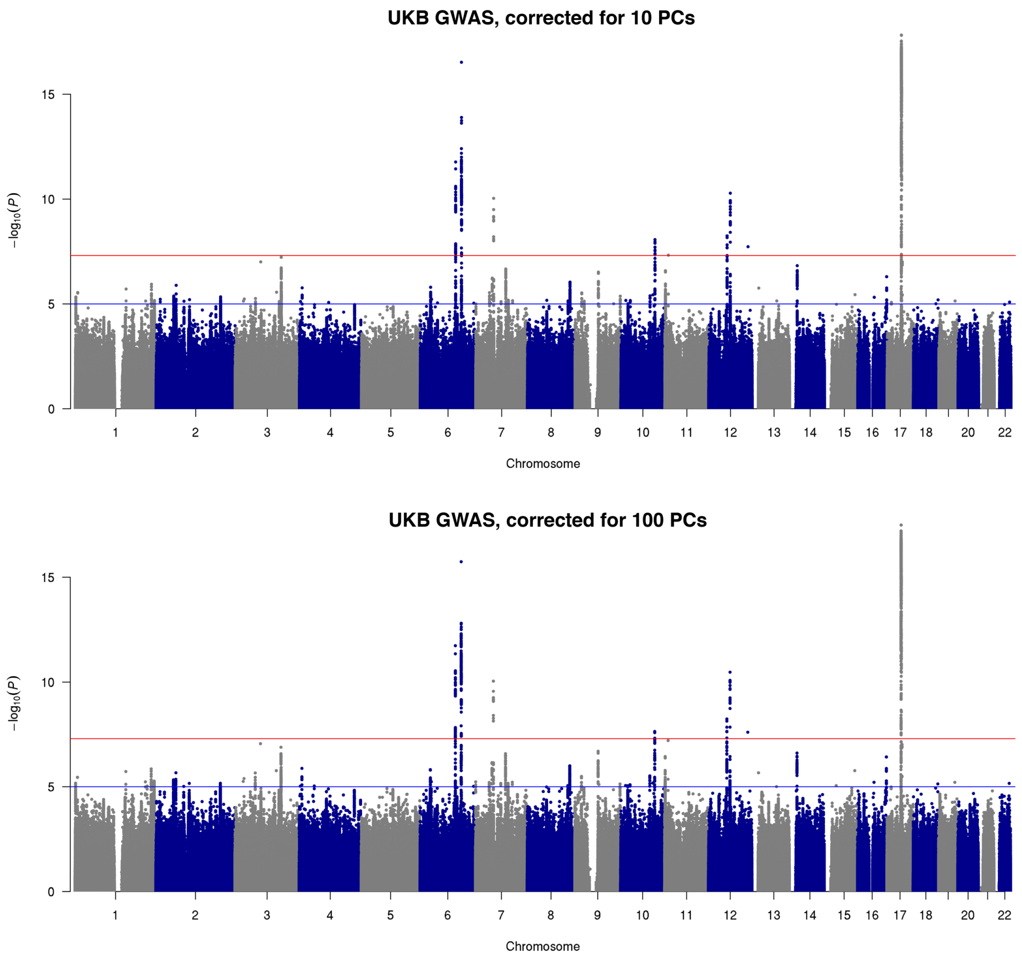

Supplementary Fig. 11. GWAS of brain volume in the UKB, using 10 vs. 100 genetic principal components. Manhattan plots showing negative $\log_{10}$-transformed *P*-values on the *y*-axis and genomic position on the *x*-axis for the BV meta-analysis correcting for 10 genomic principal components (PC; top panel) or 100 genomic PCs (bottom panel) (see **Supplementary Note 7**). The red line corresponds to the Bonferroni significance threshold of $P < 5\times10^{-8}$, while the blue line indicates the suggestive threshold of $P < 1\times10^{-5}$.

Supplementary Table 1. Meta-analyzed cohorts

Overview of the studies that were included in the meta-analysis of BV. PMID: Pubmed ID of the original publication of the results; total N=largest per variant N reported in the published summary statistics. For a complete overview of each of the individual cohorts included in the ENIGMA and head circumference GWAS, see the original publication.

Cohort	PMID	Population	total N
**UK Biobank**	-	Adults	**17,062**
**ENIGMA2 (ENIGMA; Hibar et al., 2015)**	25607358	Children + Adults	**11,373**
**Head circumference GWAS (Max Planck Institute; MPI; Haworth et al., 2019)**	30664637	Children + Adults	**18,881**
**Total**			**47,316**

Supplementary Table 2. Heritability enrichment in functional variant categories.

Enrichment analysis was performed using LD Score regression. Enrichment was calculated by dividing the proportion of heritability (h2) by the proportion of SNPs in the category. Annotations were obtained from the LD Score website (https://github.com/bulik/ldsc). Significant enrichment after Bonferroni correction (P<0.05/28) are highlighted in bold. UTR = untranslated region; TSS = transcription start site; DGF = digital genomic footprinting; CTCF = CCCTC binding factor; DHS = DNase hypersensitivity site; TFBS = transcription factor binding site.

Category	Proportion SNPs	Proportion h2	Proportion h2 SE	Enrichment	Enrichment SE	*P*
**SuperEnhancer (Hnisz)**	**0.1684**	**0.4268**	**0.0398**	**2.5344**	**0.2364**	**1.17E-08**
**H3K27ac (Hnisz)**	**0.3912**	**0.7100**	**0.0615**	**1.8152**	**0.1572**	**6.29E-07**
**Conserved (Lindblad-**	**0.0261**	**0.3925**	**0.0713**	**15.0582**	**2.7357**	**1.19E-06**
**H3K9ac (Trynka)**	**0.1261**	**0.6280**	**0.1040**	**4.9797**	**0.8244**	**1.30E-06**
**H3K4me3 (Trynka0**	**0.1333**	**0.4971**	**0.0953**	**3.7289**	**0.7152**	**9.57E-05**
**TSS (Hoffman)**	**0.0182**	**0.2020**	**0.0561**	**11.0884**	**3.0793**	**1.24E-03**
**H3K4me1 peaks (Trynka)**	**0.1713**	**0.7822**	**0.1955**	**4.5655**	**1.1411**	**1.59E-03**
**H3K9ac peaks (Trynka)**	**0.0388**	**0.4006**	**0.1151**	**10.3332**	**2.9682**	**1.74E-03**
H3K4me1 (Trynka)	0.4266	0.7841	0.1362	1.8381	0.3194	8.10E-03
Coding (UCSC)	0.0147	0.1486	0.0498	10.1360	3.3997	8.25E-03
Intron (UCSC)	0.3875	0.5167	0.0522	1.3336	0.1348	1.22E-02
DGF (ENCODE)	0.1376	0.5602	0.1844	4.0715	1.3398	2.15E-02
Repressed (Hoffman)	0.4612	0.1546	0.1318	0.3352	0.2858	2.23E-02
3-UTR (UCSC)	0.0111	0.0898	0.0358	8.1272	3.2404	2.95E-02
Transcribed (Hoffman)	0.3454	0.6263	0.1301	1.8131	0.3767	3.02E-02
Promoter (UCSC)	0.0312	0.1491	0.0560	4.7860	1.7962	3.08E-02
H3K27ac (PGC2)	0.2695	0.4672	0.1149	1.7339	0.4262	7.95E-02
Fetal DHS (Trynka)	0.0848	0.2934	0.1304	3.4618	1.5383	1.14E-01
Promoter-flanking	0.0084	0.0709	0.0440	8.4075	5.2181	1.52E-01
TFBS (ENCODE)	0.1325	0.3360	0.1584	2.5367	1.1962	1.99E-01
H3K4me3 peaks (Trynka)	0.0418	0.1600	0.0922	3.8285	2.2066	2.04E-01
5-UTR (UCSC)	0.0054	0.0368	0.0337	6.7791	6.2130	3.49E-01
WeakEnhancer (Hoffman)	0.0211	-0.0245	0.0696	-1.1617	3.3007	5.10E-01
Enhancer (Hoffman)	0.0633	0.0082	0.0955	0.1288	1.5089	5.64E-01
DHS peaks (Trynka)	0.1118	0.1973	0.1561	1.7657	1.3968	5.84E-01
DHS (Trynka)	0.1678	0.2291	0.1674	1.3659	0.9979	7.14E-01
CTCF (Hoffman)	0.0238	0.0049	0.0770	0.2066	3.2333	8.06E-01
Enhancer (Andersson)	0.0043	0.0096	0.0343	2.2141	7.9121	8.78E-01

Supplementary Table 3. Genetic correlations between intracranial volume and previous GWAS studies. Genetic correlations were calculated using LD Score regression based on publicly available GWAS summary statistics. Traits are sorted from negative to positive. Significant genetic correlations after Bonferroni correction are highlighted in bold (P<0.05/25). Pubmed = Pubmed article ID; rg = genetic correlation as computed with LDSC; SE = standard error of rg; Z = Z-statistic; P = P-value.

Trait	Author	Pubmed	Sample size	rg	SE	Z	*P*
**ADHD**	**Demontis et al. 2019**	**30478444**	**55,374**	**-0.1669**	**0.0467**	**-3.5707**	**3.56E-04**
Depressive symptoms	Okbay et al. 2016	27089181	161,460	-0.1288	0.0450	-2.8649	4.17E-03
**Insomnia**	**Jansen et al. 2019**	**30804565**	**1,331,010**	**-0.1226**	**0.0270**	**-4.5350**	**5.76E-06**
Major depression	Wray et al. 2018	29700475	480,359	-0.1094	0.0414	-2.6438	8.20E-03
**Neuroticism**	**Nagel et al. 2018**	**29942085**	**449,484**	**-0.0998**	**0.0285**	**-3.5001**	**4.65E-04**
Cigarettes per day	Liu et al. 2019	30643251	263,954	-0.0897	0.0317	-2.8316	4.63E-03
Coronary artery disease	Nikpay et al. 2015	26343387	184,305	-0.0821	0.0480	-1.7114	8.70E-02
Anxiety	Otowa et al. 2016	26857599	17,310	-0.0777	0.1111	-0.6989	4.85E-01
Type II diabetes	Scott et al. 2012	28566273	159,208	-0.0617	0.0439	-1.4065	1.60E-01
Schizophrenia	Ripke et al. 2013	25056061	77,096	-0.0414	0.0326	-1.2716	2.04E-01
Anorexia nervosa	Boraska et al. 2014	24514567	17,767	-0.0119	0.0443	-0.2685	7.88E-01
Epilepsy	Krause et al. 2018	30531953	34,889	-0.0028	0.0731	-0.0386	9.69E-01
Alzheimer	Jansen et al. 2018	24162737	466,252	0.0084	0.0676	0.1243	9.01E-01
Height	Yengo et al. 2018	25282103	693.529	0.0113	0.0282	0.4009	6.88E-01
Waist-hip ratio	Shungin et al. 2015	25673412	212,244	0.0353	0.0352	1.0036	3.16E-01
Alcoholic drinks per week	Liu et al. 2019	30643251	537,349	0.0509	0.0327	1.5544	1.20E-01
Bipolar disorder	Stahl et al. 2018	https://www.biorxiv.org/content/10.1101/173062v4	51,710	0.0612	0.0379	1.6161	1.06E-01
Multiple sclerosis	Patsopoulos et al. 2017	https://www.biorxiv.org/content/10.1101/143933v1	41,505	0.0668	0.0577	1.1561	2.48E-01
Subjective well-being	Okbay et al. 2016	27089181	298,420	0.0761	0.0574	1.3258	1.85E-01
Asthma	Gabriel et al. 2007	17611496	242,569	0.0790	0.0352	2.2443	2.48E-02
Autism spectrum disorder	Anney et al. 2017	28540026	15,954	0.1558	0.0652	2.3895	1.69E-02
**BMI**	**Yengo et al. 2018**	**25282103**	**681,275**	**0.1721**	**0.0283**	**6.0780**	**1.22E-09**
**Intelligence**	**Savage et al. 2018**	**29942086**	**269,867**	**0.2107**	**0.0310**	**6.8084**	**9.87E-12**
**Educational attainment**	**Lee et al. 2018**	**30038396**	**766,345**	**0.2248**	**0.0267**	**8.4258**	**3.58E-17**
**Birth weight**	**Horikoshi et al. 2013**	**23202124**	**26,836**	**0.2647**	**0.0686**	**3.8607**	**1.13E-04**

Supplementary Table 4. Results from conditional gene set analyses.

Conditional gene set results for all gene sets that were significant for brain volume (BV) and/or intelligence. The latter two columns show P-values of association derived from analyses were BV results were conditioned on (the gene-based Z statistics of) intelligence and vice versa. Gene sets that were significantly associated are printed in bold font.

	P-value			
**Gene set**	**BV**	**IQ**	**BV cond. on intelligence**	**Intelligence cond. on BV**
BIOCARTA_ERBB3_PATHWAY	**3.37E-06**	2.95E-01	**4.79E-06**	4.24E-01
BIOCARTA_PTEN_PATHWAY	**1.84E-06**	1.23E-01	**3.67E-06**	2.19E-01
GO_CELL_GROWTH	**1.29E-06**	5.90E-02	7.01E-06	1.14E-01
GO_CENTRAL_NERVOUS_SYSTEM_NEURON_DIFFERENTIATION	7.81E-02	**2.22E-07**	1.23E-01	**4.41E-07**
GO_COMMISSURAL_NEURON_AXON_GUIDANCE	2.84E-02	**2.27E-07**	6.06E-02	**4.41E-07**
GO_GLAND_DEVELOPMENT	**8.16E-08**	4.21E-02	**3.97E-07**	6.58E-02
GO_GROWTH	**7.96E-09**	4.33E-02	**1.01E-07**	9.25E-02
GO_MESENCHYME_DEVELOPMENT	**8.53E-08**	2.96E-01	**1.34E-07**	3.85E-01
GO_NEUROGENESIS	1.60E-04	**7.55E-08**	1.70E-03	**2.79E-07**
GO_NEURON_DEVELOPMENT	1.02E-02	**8.07E-08**	5.38E-02	**3.04E-07**
GO_NEURON_DIFFERENTIATION	1.09E-03	**4.18E-10**	9.45E-03	**1.74E-09**
GO_POSITIVE_REGULATION_OF_NERVOUS_SYSTEM_DEVELOPMENT	2.06E-02	**3.41E-06**	5.75E-02	**4.15E-06**
GO_POSITIVE_REGULATION_OF_POSTTRANSCRIPTIONAL_GENE_SILENCING	**2.53E-06**	1.68E-01	**3.49E-06**	2.30E-01
GO_POSITIVE_REGULATION_OF_PRODUCTION_OF_MIRNAS_INVOLVED_IN_GENE_SILENCING_BY_MIRNA	**2.16E-06**	4.11E-01	**2.14E-06**	4.98E-01
GO_REGULATION_OF_ARTERY_MORPHOGENESIS	**3.08E-06**	3.33E-01	**3.50E-06**	4.39E-01
GO_REGULATION_OF_GROWTH	**1.45E-07**	1.13E-01	**8.64E-07**	1.80E-01
GO_REGULATION_OF_MUSCLE_ORGAN_DEVELOPMENT	**2.50E-06**	2.31E-01	**3.44E-06**	3.14E-01
GO_REGULATION_OF_NERVOUS_SYSTEM_DEVELOPMENT	6.92E-03	**1.82E-06**	2.45E-02	**2.98E-06**
GO_REGULATION_OF_NEURON_DIFFERENTIATION	1.16E-02	**3.70E-06**	3.80E-02	7.70E-06
GO_SYNAPTIC_MEMBRANE	7.87E-01	**2.57E-06**	8.92E-01	1.39E-05
KEGG_ENDOMETRIAL_CANCER	**2.28E-07**	1.13E-01	**3.61E-07**	1.90E-01
REACTOME_DISEASES_OF_SIGNAL_TRANSDUCTION	**1.67E-06**	1.92E-01	**3.12E-06**	2.64E-01
REACTOME_ESTROGEN_DEPENDENT_NUCLEAR_EVENTS_DOWNSTREAM_OF_ESR_MEMBRANE_SIGNALING	**1.48E-06**	1.59E-01	6.94E-06	2.39E-01
REACTOME_FORMATION_OF_SENESCENCE_ASSOCIATED_HETEROCHROMATIN_FOCI_SAHF	**1.05E-06**	1.69E-01	**1.05E-06**	2.13E-01
REACTOME_GRB7_EVENTS_IN_ERBB2_SIGNALING	**1.96E-06**	3.47E-01	3.56E-05	4.56E-01
REACTOME_IRS_ACTIVATION	**1.43E-06**	5.65E-01	**1.28E-06**	7.10E-01
REACTOME_PI3K_AKT_SIGNALING_IN_CANCER	**1.53E-07**	1.76E-01	**4.32E-07**	2.59E-01

Supplementary Table 5. Enrichment of 92 overlapping genes in significant gene sets.

Results from Fisher exact tests for enrichment of the 92 genes identified for both BV and intelligence in all gene sets that were significant for brain volume (BV) and/or intelligence (Odds ratio = (overlapping genes included in the set/overlapping genes not included in the set) / (non-overlapping genes included in the set /non-overlapping genes not included in the set)). None of the enrichment tests were significant using a Bonferroni-corrected P-value threshold: P < 1.85e-3 (0.05/27) .

Gene set	Overlapping genes included in gene set	Non-overlapping genes included in gene set	Overlapping genes not included in gene set	Non-overlapping genes not included in gene set	Odds ratio	*P*-value
REACTOME_ESTROGEN_DEPENDENT_NUCLEAR_EVENTS_DOWNSTREAM_OF_ESR_MEM[illegible]	2	21	90	18056	19.09	5.99E-03
REACTOME_PI3K_AKT_SIGNALING_IN_CANCER	3	97	89	17980	6.25	1.43E-02
GO_CELL_GROWTH	6	434	86	17643	2.84	2.43E-02
BIOCARTA_ERBB3_PATHWAY	1	4	91	18073	49.48	2.51E-02
REACTOME_GRB7_EVENTS_IN_ERBB2_SIGNALING	1	4	91	18073	49.48	2.51E-02
GO_REGULATION_OF_MUSCLE_ORGAN_DEVELOPMENT	3	129	89	17948	4.69	2.95E-02
GO_CENTRAL_NERVOUS_SYSTEM_NEURON_DIFFERENTIATION	3	171	89	17906	3.53	5.84E-02
BIOCARTA_PTEN_PATHWAY	1	17	91	18060	11.67	8.74E-02
GO_GROWTH	7	908	85	17169	1.56	1.81E-01
KEGG_ENDOMETRIAL_CANCER	1	49	91	18028	4.04	2.24E-01
REACTOME_DISEASES_OF_SIGNAL_TRANSDUCTION	3	369	89	17708	1.62	2.91E-01
GO_NEUROGENESIS	9	1507	83	16570	1.19	3.60E-01
GO_GLAND_DEVELOPMENT	3	419	89	17658	1.42	3.61E-01
GO_REGULATION_OF_GROWTH	4	626	88	17451	1.27	3.96E-01
GO_REGULATION_OF_NERVOUS_SYSTEM_DEVELOPMENT	5	853	87	17224	1.16	4.40E-01
GO_NEURON_DIFFERENTIATION	7	1268	85	16809	1.09	4.69E-01
GO_REGULATION_OF_NEURON_DIFFERENTIATION	3	610	89	17467	0.97	6.04E-01
GO_NEURON_DEVELOPMENT	5	1032	87	17045	0.95	6.09E-01
GO_POSITIVE_REGULATION_OF_NERVOUS_SYSTEM_DEVELOPMENT	2	496	90	17581	0.79	7.22E-01
GO_MESENCHYME_DEVELOPMENT	1	255	91	17822	0.77	7.30E-01
REACTOME_FORMATION_OF_SENESCENCE_ASSOCIATED_HETEROCHROMATIN_FOCI_S[illegible]	0	17	92	18060	0.00	1.00E+00
REACTOME_IRS_ACTIVATION	0	5	92	18072	0.00	1.00E+00
GO_POSITIVE_REGULATION_OF_POSTTRANSCRIPTIONAL_GENE_SILENCING	0	23	92	18054	0.00	1.00E+00
GO_POSITIVE_REGULATION_OF_PRODUCTION_OF_MIRNAS_INVOLVED_IN_GENE_SILE[illegible]	0	10	92	18067	0.00	1.00E+00
GO_REGULATION_OF_ARTERY_MORPHOGENESIS	0	3	92	18074	0.00	1.00E+00
GO_SYNAPTIC_MEMBRANE	0	404	92	17673	0.00	1.00E+00
GO_COMMISSURAL_NEURON_AXON_GUIDANCE	0	12	92	18065	0.00	1.00E+00

Supplementary References

1. Yang, J. *et al.* Genomic inflation factors under polygenic inheritance. *Eur. J. Hum. Genet.* **19**, 807–812 (2011).
2. Epstein, H. T. & Epstein, E. B. The Relationship between Brain Weight and Head Circumference from Birth to Age 18 Years. *Am. J. Phys. Anthr.* **48**, 471–474 (1978).
3. Bartholomeusz, H. H., Courchesne, E. & Karns, C. M. Relationship Between Head Circumference and Brain Volume in Healthy Normal Toddlers , Children , and Adults. *Neuropediatrics* **33**, 239–241 (2002).
4. Haworth, S. *et al.* Low-frequency variation in TP53 has large effects on head circumference and intracranial volume. *Nat. Commun.* **10**, 1–16 (2019).
5. Hibar, D. P. *et al.* Common genetic variants influence human subcortical brain structures. *Nature* **520**, 224–229 (2015).
6. Willer, C. J., Li, Y., Abecasis, G. R. & Overall, P. METAL: fast and efficient meta-analysis of genomewide association scans. *Bioinformatics* **26**, 2190–2191 (2010).
7. Bulik-Sullivan, B. K. *et al.* LD Score regression distinguishes confounding from polygenicity in genome-wide association studies. *Nat. Genet.* **47**, 291–295 (2015).
8. Watanabe, K., Taskesen, E., Bochoven, A. van & Posthuma, D. Functional mapping and annotation of genetic associations with FUMA. *Nat. Commun.* **8**, 1–11 (2017).
9. Martoglio, B. & Golde, T. E. Intramembrane-cleaving aspartic proteases and disease: presenilins, signal peptide peptidase and their homologs. *Hum. Mol. Genet.* **12**, R201–R206 (2003).
10. Müller, S. A., Scilabra, S. D. & Lichtenthaler, S. F. Proteomic substrate identification for membrane proteases in the brain. *Front. Mol. Neurosci.* **9**, 96 (2016).
11. Papadopoulou, A. A. *et al.* Signal Peptide Peptidase-Like 2c (SPPL2c) impairs vesicular transport and cleavage of SNARE proteins. *EMBO Rep.* e46451 (2019).
12. Burré, J. *et al.* α-Synuclein promotes SNARE-complex assembly in vivo and in vitro. *Science (80-.).* **329**, 1663–1667 (2010).
13. Maccioni, R. B. & Cambiazo, V. Role of microtubule-associated proteins in the control of microtubule assembly. *Physiol. Rev.* **75**, 835–864 (1995).
14. Myers, A. J. *et al.* The H1c haplotype at the MAPT locus is associated with Alzheimer's disease. *Hum. Mol. Genet.* **14**, 2399–2404 (2005).
15. Coppola, G. *et al.* Evidence for a role of the rare p. A152T variant in MAPT in increasing the risk for FTD-spectrum and Alzheimer's diseases. *Hum. Mol. Genet.* **21**, 3500–3512 (2012).
16. Desikan, R. S. *et al.* Genetic overlap between Alzheimer's disease and Parkinson's disease at the MAPT locus. *Mol. Psychiatry* **20**, 1588 (2015).
17. de Leeuw, C. A., Mooij, J. M., Heskes, T. & Posthuma, D. MAGMA: Generalized gene-set analysis

of GWAS data. *PLoS Comput. Biol.* **11**, 1–19 (2015).

18. Brunet, A. *et al.* Akt promotes cell survival by phosphorylating and inhibiting a Forkhead transcription factor. *Cell* **96**, 857–868 (1999).

19. Medema, R. H., Kops, G. J. P. L., Bos, J. L. & Burgering, B. M. T. AFX-like Forkhead transcription factors mediate cell-cycle regulation by Ras and PKB through p27 kip1. *Nature* **404**, 782 (2000).

20. Carraway, K. L. *et al.* The erbB3 gene product is a receptor for heregulin. *J. Biol. Chem.* **269**, 14303–14306 (1994).

21. Prigent, S. A. & Gullick, W. J. Identification of c-erbB-3 binding sites for phosphatidylinositol 3′-kinase and SHC using an EGF receptor/c-erbB-3 chimera. *EMBO J.* **13**, 2831–2841 (1994).

22. Liberzon, A. *et al.* Molecular signatures database (MSigDB) 3.0. *Bioinformatics* **27**, 1739–1740 (2011).

23. Accili, D. & Arden, K. C. FoxOs at the crossroads of cellular metabolism, differentiation, and transformation. *Cell* **117**, 421–426 (2004).

24. de Leeuw, C. A., Stringer, S., Dekkers, I. A., Heskes, T. & Posthuma, D. Conditional and interaction gene-set analysis reveals novel functional pathways for blood pressure. *Nat. Commun.* **9**, 1–13 (2018).
