# Supplementary material for: Genome-wide meta-analysis of brain volume identifies genomic loci and genes shared with intelligence
Source: Nat Commun. 2020 Nov 5;11:5606. doi: 10.1038/s41467-020-19378-5 (PMC7644755; doi:10.1038/s41467-020-19378-5)
Supplement: Supplementary file 3 — Reporting Summary [file 41467_2020_19378_MOESM3_ESM.pdf]

nature research

Corresponding author(s): Danielle Posthuma

Last updated by author(s): Aug 20, 2020

Reporting Summary

Nature Research wishes to improve the reproducibility of the work that we publish. This form provides structure for consistency and transparency in reporting. For further information on Nature Research policies, see our Editorial Policies and the Editorial Policy Checklist.

Statistics

For all statistical analyses, confirm that the following items are present in the figure legend, table legend, main text, or Methods section.

n/a	Confirmed	
[ ]	[x]	The exact sample size (*n*) for each experimental group/condition, given as a discrete number and unit of measurement
[x]	[ ]	A statement on whether measurements were taken from distinct samples or whether the same sample was measured repeatedly
[ ]	[x]	The statistical test(s) used AND whether they are one- or two-sided<br>*Only common tests should be described solely by name; describe more complex techniques in the Methods section.*
[ ]	[x]	A description of all covariates tested
[ ]	[x]	A description of any assumptions or corrections, such as tests of normality and adjustment for multiple comparisons
[ ]	[x]	A full description of the statistical parameters including central tendency (e.g. means) or other basic estimates (e.g. regression coefficient) AND variation (e.g. standard deviation) or associated estimates of uncertainty (e.g. confidence intervals)
[ ]	[x]	For null hypothesis testing, the test statistic (e.g. *F*, *t*, *r*) with confidence intervals, effect sizes, degrees of freedom and *P* value noted<br>*Give P values as exact values whenever suitable.*
[x]	[ ]	For Bayesian analysis, information on the choice of priors and Markov chain Monte Carlo settings
[x]	[ ]	For hierarchical and complex designs, identification of the appropriate level for tests and full reporting of outcomes
[ ]	[x]	Estimates of effect sizes (e.g. Cohen's *d*, Pearson's *r*), indicating how they were calculated

Our web collection on statistics for biologists contains articles on many of the points above.

Software and code

Policy information about availability of computer code

Data collection	No software was used for data collection purposes.
Data analysis	The following standard software packages were used for the analyses described in the current manuscript:<br>MAGMA: In-house developed software (de Leeuw et al., 2015) was used to conduct gene and gene-set analysis.<br>FUMA: In-house developed online platform for functional annotation of GWAS results (Watanabe et al., 2017).<br>PLINK1.9: Open-source software (Chang et al., 2015) used for performing genome-wide association analysis.<br>GSMR: Generalized summary-data-based Mendelian randomization (Zhu et al., 2018) was used for Mendelian randomization analysis.

For manuscripts utilizing custom algorithms or software that are central to the research but not yet described in published literature, software must be made available to editors and reviewers. We strongly encourage code deposition in a community repository (e.g. GitHub). See the Nature Research guidelines for submitting code & software for further information.

Data

Policy information about availability of data

All manuscripts must include a data availability statement. This statement should provide the following information, where applicable:

- Accession codes, unique identifiers, or web links for publicly available datasets
- A list of figures that have associated raw data
- A description of any restrictions on data availability

In the present study we made use of the publicly available gene expression data at GTEx Consortium websites and from the Allen Human Brain Atlas, scRNA-seq data deposited at DropViz, as well as GWAS summary statistics. All URLs are provided in the manuscript.

Field-specific reporting

Please select the one below that is the best fit for your research. If you are not sure, read the appropriate sections before making your selection.

- [x] Life sciences
- [ ] Behavioural & social sciences
- [ ] Ecological, evolutionary & environmental sciences

For a reference copy of the document with all sections, see nature.com/documents/nr-reporting-summary-flat.pdf

Life sciences study design

All studies must disclose on these points even when the disclosure is negative.

Sample size	We made use of data collected by external sources (UK biobank, ENIGMA consortium, Max Planck Institute). For all samples the sample size consists of all individuals that remain after quality control of the data and exclusion of withdrawn subjects. Detailed information on the samples used, as well as the exclusion/inclusion criteria, are provided in the Online Methods (sections: Samples & phenotypes, Genotyping, imputation & quality control, GWAS of total brain volume).
Data exclusions	See Online Methods.<br>For UKB data: we excluded participants from further analyses if they had excessive missing phenotypic data (section: Phenotype assessment), did not pass standard quality control or withdrew their consent to participate in the UK biobank study (section: Genotyping and imputation).
Replication	We used a meta-analytic approach, which inherently evaluates the combined evidence for significant association across samples. We explicitly examined the concordance of effects across the three individual cohorts.
Randomization	NA
Blinding	NA

Reporting for specific materials, systems and methods

We require information from authors about some types of materials, experimental systems and methods used in many studies. Here, indicate whether each material, system or method listed is relevant to your study. If you are not sure if a list item applies to your research, read the appropriate section before selecting a response.

Materials & experimental systems

n/a	Involved in the study	
x		Antibodies
x		Eukaryotic cell lines
x		Palaeontology and archaeology
x		Animals and other organisms
	x	Human research participants
x		Clinical data
x		Dual use research of concern

Methods

n/a	Involved in the study	
x		ChIP-seq
x		Flow cytometry
	x	MRI-based neuroimaging

Human research participants

Policy information about studies involving human research participants

Population characteristics	We utilized data collected previously by UK biobank. All individuals included in the study provided informed consent, and the study was approved by the concerned ethical committee.
Recruitment	See above (and in Methods section of the manuscript)
Ethics oversight	NHS Health Research Authority provided ethics approval for the UKB study

Note that full information on the approval of the study protocol must also be provided in the manuscript.

Magnetic resonance imaging

Experimental design

Design type	No dynamic MRI data collection was performed, only cross-sectional structural brain imaging
Design specifications	not applicable

Behavioral performance measures | not applicable

Acquisition

Imaging type(s)	Structural brain imaging (T1-weighted sequence)
Field strength	Structural brain imaging (T1-weighted sequence)
Sequence & imaging parameters	Structural T1-weighted sequence: 3D MPRAGE gradient-echo, FOV: 1x1x1 mm, matrix size: 208x256x256, resolution: 1x1x1 mm (1mm slice thickness), orientation: sagittal, TI/TR : 880/2000 ms
Area of acquisition	Whole brain scan

Diffusion MRI ☐ Used ☒ Not used

Preprocessing

Preprocessing software	FSL (FMRIB Software Library)
Normalization	linear alignment and non-linear warping to the MNI152 brain template (FSL FLIRT/FNIRT)
Normalization template	standard MNI152 brain template
Noise and artifact removal	gradient distortion correction, bias field correction
Volume censoring	defacing, brain extraction (FSL BET), segmentation and extraction of brain structure volumes (FSL FAST software)

Statistical modeling & inference

Model type and settings	Extracted brain volume from structural MRI data was used in linear regression models in GWAS, no statistical modeling was performed on the imaging data itself
Effect(s) tested	*Define precise effect in terms of the task or stimulus conditions instead of psychological concepts and indicate whether ANOVA or factorial designs were used.*

Specify type of analysis: ☒ Whole brain ☐ ROI-based ☐ Both

Statistic type for inference (See Eklund et al. 2016)	standard linear regression model for testing associations between SNPs and brain volume phenotypes derived from the T1-weighted scan
Correction	Bonferroni correction for the number of independent variants that were tested in association with ICV (P<5x10-8)

Models & analysis

n/a	Involved in the study
☒	☐ Functional and/or effective connectivity
☒	☐ Graph analysis
☒	☐ Multivariate modeling or predictive analysis
