# Supplementary material for: Genome-wide meta-analysis of brain volume identifies genomic loci and genes shared with intelligence
Source: Nat Commun. 2020 Nov 5;11:5606. doi: 10.1038/s41467-020-19378-5 (PMC7644755; doi:10.1038/s41467-020-19378-5)
Supplement: Supplementary file 4 — Description of Additional Supplementary Files [file 41467_2020_19378_MOESM4_ESM.pdf]

Description of Additional Supplementary Files

File name: Supplementary Data 1
Description: LD Score heritability estimates of individual cohorts and meta-analyses, and of between-cohort genetic correlations based on summary statistics. n = sample size; intercept = LD Score intercept; rg = genetic correlation; SE = standard error of the genetic correlation.

File name: Supplementary Data 2
Description: Genomic loci and lead variants in the GWAS meta-analysis of brain volume. Locus# = locus number; lead variant rsID = variant ID of the lead variant within the locus; Chr = chromosome; BP = base pair position lead SNP; A1 = effect allele linked to the z-score; A2 = non-effect allele; Z = z-score from the meta-analysis; P = P-value; Dir = direction of effect in UKB/ENIGMA/HC-GWAS (MPI data); Start = locus BP start position of the locus; End = stop position of the locus; n ind Sign variants = number of independent significant variants within the locus; IndSigvars = rsIDs of the independent significant variants in the locus. Loci marked with an asterisk were identified previously by Adams et al. (2016). See Methods for details on the locus definition. The locus in red font was not considered in the current study, since we have low confidence in it (see Supplementary Note 3).

File name: Supplementary Data 3
Description: Genome-wide significant variants in the GWAS meta-analysis of brain volume and for individual cohorts. In the meta-analysis, variant P-values were computed using a weighted z-score method in METAL. See Methods for details on how the P-values for the ENIGMA and HC-GWAS (Haworth et al., 2019) data were calculated. rsID = rs number of the variant; Unique ID = ID variable formatted as 'chromosome:base pair position:alleles in alphabetic order'; CHR = Chromosome; BP = base pair position; A1 = effect allele; A2 = non-effect allele; N = per variant sample size; Z = Z-statistic; Beta = Beta regression coefficient; P-value = P-value; DIR = direction of variant effect in UKB/ENIGMA/HC-GWAS cohort; MAF UKB = minor allele frequency of the variant in the UKB data; INFO UKB = imputation quality of the variant in the UKB data.

File name: Supplementary Data 4
Description: Functional annotation results in FUMA of candidate variants obtained in the brain volume GWAS. Annotation was performed in FUMA using the SNP-to-gene annotation functions, which carries out annotation in ANNOVAR. Candidate variants were defined as having a GWAS P-value of P < 1e-05 and being in high LD (>0.6) with on the independent significant variants. Locus = genomic locus in which the variant is located; CHR = Chromosome; BP = base pair position; MAF = minor allele frequency in 1000 Genomes; NEA = Non-effect allele; EA = Effect allele; GWAS P = P-value in the GWAS; Z-score = z-score provided in the input GWAS summary statistics file if available; r2 = LD between the variant and the independent significant variant; Ind. Sig. var = independent significant variant; Nearest gene = symbol of gene located most closely to the variant; Annotation = genomic region annotated by ANNOVAR; CADD score = Combined Annotation Dependent Score, the higher the CADD score, the more deleterious the variant is likely to be; RBD score = Regulome DB score categories representing biological evidence to be a regulatory element; Min. Chrom. state = The minimum 15-core chromatin state across 127 tissue/cell type; Common Chrom. state = The most common 15-core chromatin state across 127 tissue/cell types.

File name: Supplementary Data 5
Description: Overview of candidate exonic nonsynonymous SNPs related to brain volume. SNP = Single nucleotide polymorphism rsID/Unique ID; Locus = Index of the genomic risk loci; CHR = chromosome number; BP = basepair position; A1 = effect allele; A2 = non-effect allele; Z = z-score in the validation GWAS meta-analysis; P = association P-value; MAF = minor allele frequency; Gene =

closest gene; CADD = CADD score; RDB = RegulomeDB score; Min. Chrom. State = The minimum 15-core chromatin state across 127 tissue/cell type; Exon = exon number of the SNP location; Shift A1/A2=amino acid shift between A1 and A2.

File name: Supplementary Data 6
Description: Genes observed in the variant-based gene-mapping of brain volume in FUMA.
Genes identified by FUMA through positional mapping, eQTL mapping and/or chromatin interaction mapping. The GWAS meta-analysis summary statistics of brain volume (UKB/ENIGMA/HC-GWAS) were used as input. Data of the ENIGMA and head circumference GWAS were corrected for height using mtCOJO (see Methods). Ensembl ID = Ensembl gene ID; Entrez ID = Entrez gene ID; HUGO = HUGO gene symbol; CHR = chromosome; Start/End = start and end position of the genomic locus; Strand = coding strand; pLI = pLI score from ExAC database. The probability of being loss-of-function intolerant. The higher the score is, the more intolerant to loss-of-function mutations the gene is; ncRVIS = non-coding residual variation intolerance score. The higher the score is, the more intolerant to non-coding variation the gene is; Mapped by = mapping method through which the gene was identified (positional = positionally mapped; eQTL = expression quantitative trait locus; CI = chromatin interaction); n Mapped=number of gene-mapping methods that implicated the gene in FUMA; Pos. Mapped variants = number of variants mapped to gene based on positional mapping (after functional filtering if parameters are given); Max. CADD score = maximum CADD score of mapped variants by positional mapping; eQTL Mapped variants = number of variants mapped to the gene based on eQTL mapping; eqtlMapminP = The minimum eQTL P-value of mapped variants; CI Mapped = "Yes" if the gene is mapped by chromatin interaction mapping, "No" otherwise; CI tissue = tissue/cell types of mapped chromatin interactions; minGwasP = The minimum P-value of mapped variants; IndSigvar = rsID of the all independent significant variants of mapped variants; Genomic Locus = Index of genomic loci where mapped variants are from.

File name: Supplementary Data 7
Description: Genes significantly associated with brain volume in the gene-based association test using MAGMA. Gene-based analyses were conducted using the GWAS meta-analysis P-values of brain volume (UKB, ENIGMA2 & HC-GWAS) as input. This table includes only genes that reached genome-wide significance (0.05/N genes = 0.05/18168 = 2.75E-06). Entrez ID/Symbol = Gene Entrez ID/symbol; CHR = chromosome; START/STOP = start/end position of the gene in base pairs; NSNP = number of SNPs in gene; NPARAM = number of relevant parameters used in the model; N = sample size; Z = Z-value for the gene, based on its P-value; P-value = gene P-value.

File name: Supplementary Data 8
Description: Genes that were identified in previous genetic association studies of brain volume. The last column indicates whether the genes was also significantly associated to BV in the current study.

File name: Supplementary Data 9
Description: Look-up of all implicated genes in the OMIM database. Genes implicated in BV were searched for in the Online Mendelian Inheritance in Man database (OMIM: https://www.omim.org/). Look-up was performed for genes that were implicated by any of the gene-mapping methods. Gene = gene symbol; CHR = chromosome number; Start/End = start/end base pair position; OMIM entry = entry number; Disease = disease associated with the gene in the OMIM database; Inheritance = inheritance pattern (AR = autosomal recessive; AD = autosomal dominant); Phenotype MIM = Phenotype ID in the OMIM database.

File name: Supplementary Data 10
Description: Gene-sets, tissue and cell-types involved in brain volume.

Gene-sets were obtained from the MSigDB gene-set database (v7.0). Tissue gene-expression data was obtained via the GTEx portal. Gene-expression in 565 distinct adult mouse brain cell-types were derived from Dropviz. Gene-set = abbreviated gene-set name; N genes = number of genes from the gene-based association analyses that were included in the gene-set; P = P-value from the competitive test; Full-name = full MSigDB gene-set name. Gene-sets tissue types and cell types passing the corrected significance thresholds (P < 3.90E-06; 0.05/(12,191 gene sets + 53 tissue types + 565 cell types) are highlighted in bold, correcting for the number of tested functional gene-sets, tissue and cell types. Results are ranked by P-value (from low to high).

File name: Supplementary Data 11
Description: Gene-sets of genes mapped by the GWAS loci of brain volume. Gene-set enrichment analysis of genes that were implicated by FUMA through positional mapping, eQTL mapping and chromatin-chromatin interactions. Gene-set enrichment significance was tested using hypergeometric tests. Curated gene sets were obtained from the MSigDB website (MSigDB v6.2). In total 10,679 gene sets were tested (C2 and C5 gene sets). Gene sets are shown that were significant after correction for multiple testing (P<0.05/10,679 = 4.68E-06). N genes = number of genes in the gene set; N overlapping = number of implicated genes for BV in the gene set; genes = gene ID of genes implicated in BV and located in the gene set; P = P-value.

File name: Supplementary Data 12
Description: Results of pairwise conditional gene-set analyses of 18 significant gene-sets for brain volume. Tested = gene-set 1 in the analysis; Conditioned on = gene-set 2 (used to condition on); P_marg = marginal P-value of association for gene-set 1 ("Tested"); P_cond = P-value resulting from the conditional analysis fo gene-set 1 on gene-set 2; Proportion of significance = scaled difference between marginal and conditional P-values, computed as: (-log10(P_cond) minus -log10(P_marg)) / -log10(P_marg). Gene-sets indicated by an asterisk are largely independent gene-sets (i.e. conditional P value remaining significant after conditioning in more than half over the conditional tests). P_cond P-values in bold highlight conditional P-values that remained significant after Bonferroni correction.

File name: Supplementary Data 13
Description: Overview of overlapping genes involved in brain volume and intelligence.
Overview of genes that were identified through FUMA and/or MAGMA to be associated to brain volume (BV; UKB/ENIGMA/HC-GWAS) as well as to intelligence. We indicate whether 1) the gene contains an exonic nonsynonymous (ExNS) SNP or a splicing SNP (see Supplementary Data 5), 2) if the gene colocalizes with an eQTL and 3) whether the gene was part of a gene set that was significantly associated to BV and/or intelligence.
Genes to prioritize are printed in blue font. These genes satisfy one or more of the following filtering conditions: 1. contain a ExNS SNP; 2. part of one or more gene sets associated to either trait (BV and/or intelligence); 3. colocalization of GWAS and eQTL signals.
MAGMA gene-based P-values printed in bold indicate P<2.75e-6 (=0.05/Ngenes). Entrez ID = Entrez ID of gene from NCBI database; HUGO symbol = gene symbol; CHR = chromosome number; Start/End = start/end basepair position of gene; Genomic locus = genomic locus ID as assigned by FUMA for BV and/or intelligence; MAGMA gene-based P-value = gene P-value resulting from MAGMA's gene-based analysis on BV/intelligence; FUMA mapping = mapping method in FUMA through which the gene was identified; ExNS = indicator of whether the gene contains an ExNS SNP in the analysis of BV/intelligence; Part of sign. gene set = indicator of whether the gene is part of one of the significant gene sets; Colocalization = tissue for which the GWAS and eQTL signals were found to colocalize.

File name: Supplementary Data 14

Description: Gene expression patterns of the 92 overlapping genes between brain volume and intelligence in 57 AHBA brain regions. Results of permutation tests for the full set of overlapping genes (n=92) and for two subsets that were identified in cluster analysis. Percentage = indicates the of permuted samples for which the average expression in a given region was higher than for the set (/cluster) of genes of interest (e.g., "Percentage" > 0.5 means decreased gene expression and < 0.5 means increased gene expression); P-value = uncorrected two-sided P-values obtained by means of permutation. P-values lower than the Bonferroni-corrected significance threshold (P < 0.00088 (=0.05/57)) are printed in bold font.

File name: Supplementary Data 15:
Description: Interaction gene-set analysis of brain volume in MAGMA. Results of interaction gene-set analysis, aimed at testing whether there were significant interaction effects of BV-related genes and gene expression (across 57 brain regions) on intelligence. After stringent Bonferroni (0.05/57) correction none of the interactions remained significant. Interaction = variables in the interaction analysis (first term is always the set of BV-related genes, second term refers to one of 57 AHBA defined brain regions).
