# Supplementary material for: Genome-wide meta-analysis of brain volume identifies genomic loci and genes shared with intelligence
Source: Nat Commun. 2020 Nov 5;11:5606. doi: 10.1038/s41467-020-19378-5 (PMC7644755; doi:10.1038/s41467-020-19378-5)
Supplement: Supplementary file 2 — Peer Review File [file 41467_2020_19378_MOESM2_ESM.pdf]

Editorial Note: This manuscript has been previously reviewed at another journal that is not operating a transparent peer review scheme. This document only contains reviewer comments and rebuttal letters for versions considered at *Nature Communications.* [Mentions of prior referee reports have been redacted.]

Reviewers' comments:

Reviewer #2 (Remarks to the Author):

The authors present a genome-wide association study of brain volume in UK Biobank and meta-analysed those results with brain volume summary statistics from ENIGMA and a GWAS of head circumference (Haworth et al. 2019). The results from the meta-analysis of brain volume was then used to examine the relationship with intelligence using publicly available summary statistics from Savage et al. 2018.
I reviewed this manuscript in a previous round of revisions at another journal and I am satisfied that the authors have dealt with all my original comments. The manuscript is considerably stronger following the previous round of revisions with the analyses clearly described and the results interpreted fairly for which the authors should be commended.
I do have a few further comments which are detailed below:

Comments

1. The use of “yet” on line 27 is superfluous
2. The authors should briefly comment on the genetic correlation of 1.25 for brain volume between UK Biobank and ENIGMA. Typically values outside of -1 to +1 can occur with low heritabilities or small sample sizes, but that doesn’t seem to be the case here
3. Of the 4,155 significant SNPs in the BV meta-analysis, 272 were indels. Although indels aren’t strictly classified as SNPs, it might be worth highlighting the inclusion of indels within the brackets on line 62.
4. On line 131, rather than state that there were “several gene sets”, please state the number
5. On lines 179-180, the authors note the close resemblance between their genetic correlations calculated using SNP data and other correlations made with twin data. These two approaches are not comparable, as twin studies typically have much higher correlations compared to SNP-based methods. I would advise that this sentence is rephrased.
6. Figures 3c and 3d are cited in the text before 3a and 3b, so potentially the order of the subplots within figure 3 need to be updated to reflect the current manuscript.
7. On lines 245-247, the authors are “unable to conclude that the set of overlapping genes is expressed in any particular cortical region”. Is it possible to conduct a power analysis to determine whether there is no effect or that the analysis is underpowered?
8. In Figure 3, there is a line of text running across some of the images. This may just be a pdf creation artefact.
9. On line 421, change “firsts” to “first”
10. Define CADD add first usage on line 493
11. For data availability, it would be useful if the authors could also make the individial height-adjusted summary stats for brain volume available for UK Biobank and ENIGMA and the height-adjusted summary stats for head circumference from Haworth et al.
12. Some of the journal volumes and page/article numbers listing in the references are incorrect or missing. For example, Savage et al. is listed as Nature Genet, 1, but should be Nature Genet, 50, 912–919. Haworth et al is missing the article number (357), as is Watanabe et al. (1826). Please check all references for completeness and accuracy.
13. In the Supplementary information lines 46-47, an r value is provided for those under 6 years of age, but not for the older age groups. Please could the r values for the older age groups be added?
14. In supplementary figure 4, the coloring of the LD for each of the zoom plots are not displaying correctly. Would it also be possible to use dots to represent each SNP rather than boxes?
15. In supplementary table 1, the sample sizes for the studies within ENIGMA (n = 13,688) don’t quite

add up to the total reported for ENIGMA (n = 11,373). The same is true for the HC study, although only differing by 3 participants. Please double check these numbers.

Reviewer #3 (Remarks to the Author):

The authors present a study examining the genetic coheritability of brain volume (BV) and intelligence (i.e., a measure of the extent to which the link between brain volume and intelligence can be explained by genetics). The authors proceed by considering a meta-analysis of the ENIGMA, UK Biobank and other consortia and by using the popular Linkage Disequilibrium Score Regression (LDSC) method for estimating genetic correlation in meta-analysis.

The authors report a genetic correlation between BV and intelligence of 0.21 (page 8, line 159). The authors also follow up with additional results such as genetic correlation between BV and other traits such as educational attainment, partitioned heritability in which GWAS are examined for enrichment in annotated areas of the genome, and discussion of involved genes.

The quality of the science in this paper is very high and the methodology is valid and well described. And the authors have replied extensively to concerns raised in a previous review, greatly improving the quality of the paper.

However, I view some of the points previously raised by reviewers as still unresolved.

Regarding comment 1 of Reviewer #3. The reviewer recommends that socioeconomic indicators be regressed out as a control. The authors respond "The main purpose of the current study is not to dissect the relation between socioeconomic factors and brain volume or intelligence. We believe this might be stigmatizing ..." I believe that it's actually the opposite that is stigmatizing. It's the failure to control for socioeconomic indicators that is stigmatizing. Imagine a SNP becomes correlated with a particular geographic region through genetic drift. Suppose that subjects living in this area have lower than average brain volume due to childhood malnutrition and also lower than average intelligence due to reduced access to education. This hypothetical SNP will appear as a contribution to the genetic coheritability of BV and intelligence, as it covaries with geography. However, this contribution is irrelevant to questions of biology or basic science. Consideration of this SNP without "dissecting the relation between socioeconomic factors and brain volume and intelligence" may be stigmatizing.

Now the question about whether or not this is confounding the authors study. The authors have now included a version of their GWAS with 100 genetic PCs (discussed in their reply to comment 12 of Reviewer #2), and we can see there's not much difference in the Manhattan plots. That indicates no population stratification. However, this functions only as a most basic control for socioeconomic status. More work is required in order to adequately control for socioeconomic indicators.

Regarding comment 8 of Reviewer #2, it's still unclear why genetic correlation between BV and intelligence is important to basic science, or how this work could lead to practical application.

Reviewer #4 (Remarks to the Author):

This is a revised version of a manuscript that investigates the specific genetic factors driving the well known phenotypic correlation between brain volume and IQ.
This study offers new information in this new meta-analysis of ~47K individuals in which 18 genomic loci (including 14 novel loci), are identified, implicating 343 genes (270 novel) and 18 biological pathways for brain volume. They identify 92 shared genes between BV and intelligence, which are mainly involved in signaling pathways regulating cell growth. The manuscript has a number of

strengths, including some important followup analyses for understanding the possible mechanisms underlying these relationships, including examination of the pathways implicated, tissue and gene-expression analysis, Mendelian randomization analysis, and partitioning of heritability.
The authors have responded in detail to many of the previous detailed questions from the reviewers, and thus my remaining comments are minor /'big picture' comments.

-That there are 143 monogenic disorders caused by high-penetrance mutations in 89 of the 343 BV-implicated genes is an interesting finding,regarding the genetic architecture of determinants of brain volume . Notably, also the higher proportion of mutation intolerance for genes implicated in both BV and intelligence (vs either trait alone) is very interesting. How different is this from what would be expected by chance?

- The analysis of regional gene expression using the AHBA , which concludes that the set of overlapping genes for BV and intelligence is not preferentially expressed in any particular cortical region, seems a bit circular, given that the association is for two highly global traits, by definition. The motivation behind this particular analysis is unclear.

-Regarding the previous comment that there was not adequate control for socioeconomic indicators, the authors indicate that they did not wish to conduct 'stigmatizing analysis'. it is ironic that the measure of intelligence used here, Spearmans' g, was used to support the eugenics movement. This is just a comment, however, probably outside the scope of this manuscript. Nevertheless, a bit more thoughtful commentary about the role of gene-environment correlation in these findings would be welcome.

Response to reviewers

Reviewers comments are reproduced in full below. Our responses are added in blue.

Reviewer #1

Remarks to the Author

The authors present a genome-wide association study of brain volume in UK Biobank and meta-analysed those results with brain volume summary statistics from ENIGMA and a GWAS of head circumference (Haworth et al. 2019). The results from the meta-analysis of brain volume was then used to examine the relationship with intelligence using publicly available summary statistics from Savage et al. 2018.
I reviewed this manuscript in a previous round of revisions at another journal and I am satisfied that the authors have dealt with all my original comments. The manuscript is considerably stronger following the previous round of revisions with the analyses clearly described and the results interpreted fairly for which the authors should be commended.
I do have a few further comments which are detailed below:

Comments

1. The use of “yet” on line 27 is superfluous
We have removed ‘yet’ from this sentence.

2. The authors should briefly comment on the genetic correlation of 1.25 for brain volume between UK Biobank and ENIGMA. Typically values outside of -1 to +1 can occur with low heritabilities or small sample sizes, but that doesn’t seem to be the case here

The out-of-bound genetic correlation is likely due to a high genetic correlation between a similar phenotype in UKB and ENIGMA2, and a high standard error of the estimate (SE=0.2). The estimate of 1.25 is within the confidence interval limits of a correlation of 1, and the r_g may thus be due to sampling variation.
To make sure the r_g of > 1 was not due to errors in the input files, we performed several checks of the GWAS summary statistics files (sample size, effect size/SE distribution, p-values), and no errors were observed. We also did not find a suspicious r_g estimate when running LDSC between these sumstats and other GWAS results (see fig. 1d and supplementary table 15).

We now comment on the possibility of observing an r_g of >1 when using LDSC:

- We have added a note in the main text to explain the finding of an r_g of > 1: *“We note that the r_g outside [-1, 1] is likely due to the unbiased r_g estimation by LDSC, the large standard error of the estimate and the high genetic correlations between these meta-analyzed cohorts”* (bottom p. 4)
- We have added a clarification in the Methods section where we describe LDSC: *“the r_g estimated by LDSC is an unbiased estimate, and may exceed [-1, 1] in case of large standard errors, and a high genetic correlation between studies”* (p. 27)

3. Of the 4,155 significant SNPs in the BV meta-analysis, 272 were indels. Although indels aren’t strictly classified as SNPs, it might be worth highlighting the inclusion of indels within the brackets on line 62.

We thank the reviewer for this comment and note that the correct number of indels is 371 and not 272 (see supplementary table 4, columns A1 and A2, 272 is likely obtained from column A2 only). As suggested, we now mention the number of indels in brackets (p. 4).
“Sample-size weighted fixed-effects meta-analysis was carried out using METAL[28] (Online Methods) resulting in 24 linkage disequilibrium (LD) independent lead variants (r^2<0.1), residing in 18 genomic loci (Fig. 1a; Supplementary Table 3; Supplementary Fig. 4), representing 4,155 genome-wide significant variants (of which 371 were indels, Supplementary Table 4)”

Since we also analyzed indels in the GWAS, we have replaced the use of ‘SNPs’ with the more general term ‘(genetic) variants’ where appropriate throughout the manuscript.

4. On line 131, rather than state that there were “several gene sets”, please state the number
We define a gene-set as largely independent when more than half (i.e. more than 8 of the 17 conditional P) of the conditional *P*-values of the gene-set conditioned on each of the other gene-sets remained statistically significant. This resulted in 9 of the 18 significant gene-sets being independent. This has now been clarified.

The following changes have been made to the manuscript to address this comment:

- We have added the description of the selection of largely independent gene-sets in the methods section (p. 26): *“Gene-sets that showed a significant conditional P-value in more than half of the conditional analyses were denoted as ‘largely independent’ gene-set associations.”*
- We have added an asterisk in the Supplementary Table 14 indicating which 9 of the 18 gene-sets were independent.

- We have modified Supplementary Fig. 9, which now includes a bar plot that shows the number of conditional *P*-values that remained significant. The results are now also mentioned in the Supplementary Results 2.4 section (p. 9): *"Since gene-sets are hierarchically structured with several gene-sets being partly nested within each other, some gene-sets may be implicated through their dependence on other gene-sets. To investigate whether the identified gene-sets were independently associated with BV, we carried out conditional gene-set analyses. Conditional gene-set analysis calculates P-values for each gene-set conditional on the association of each of the other gene-sets. These analyses showed that 9 of the 18 gene-sets were largely independent signals (defined as conditional P-values that remained significant in more than half (i.e. more than 8) of the conditional analyses, Supplementary Fig. 9, Supplementary Table 14). The gene-sets with the largest number of conditional P-values remaining significant were REACTOME: IRS activation (n=16), REACTOME: Formation of senescence-associated heterochromatin foci SAHF (n=16), GO: Growth (n=15), suggesting that these gene-set were the most independent from the other implicated gene-sets.*

5. On lines 179-180, the authors note the close resemblance between their genetic correlations calculated using SNP data and other correlations made with twin data. These two approaches are not comparable, as twin studies typically have much higher correlations compared to SNP-based methods. I would advise that this sentence is rephrased.
We have now rephrased this sentence and no longer compare the GWAS and twin study estimates, we have removed the twin genetic correlation estimates from this section. We only mention that a significant genetic correlation based on LD Score regression confirms a significant genetic correlation between brain volume and intelligence from twin studies (p. 8):
*"Our analyses confirmed the previously established genetic overlap between BV and intelligence: the estimated r_g between BV and intelligence was 0.24 (**Supplementary Tables 2, 15**), in line with prior evidence of shared genetic factors between intelligence and gray matter volume and white matter volume obtained from twin studies"*

6. Figures 3c and 3d are cited in the text before 3a and 3b, so potentially the order of the subplots within figure 3 need to be updated to reflect the current manuscript.
Thank you for pointing this out, the order of the sub plots in figure 3 has been updated to match the order of the results section (in the current version fig 3a-b: cross-trait lead SNP qq-plot; fig. 3c-d: MR analyses).

7. On lines 245-247, the authors are "unable to conclude that the set of overlapping genes is expressed in any particular cortical region". Is it possible to conduct a power analysis to determine whether there is no effect or that the analysis is underpowered?
Previous work from our group discussed the power of gene-set analyses in a recent *Nature Rev Genet* article (see de Leeuw et al., 2016), where simulation analyses showed that the power of gene-set analyses largely depends on the size of the gene-set (here: the set of overlapping genes), the sample size of the study, the (background) heritability of the trait of interest, and gene-set specific heritability. Although the gene-expression analyses are slightly different (comparison of gene-expression of genes within a gene-set to outside the set, instead of gene-associations), similar principals apply here.
We note that these analyses showed no association with expression in particular cortical areas more than average. We have rephrased this sentence (p. 10):
"Based on these results, we are unable to conclude that the set of overlapping genes is predominantly expressed in any particular cortical region compared to other brain regions.

8. In Figure 3, there is a line of text running across some of the images. This may just be a pdf creation artefact.
Thank you, the figure has been corrected.

9. On line 421, change "firsts" to "first"
This has been corrected.

10. Define CADD add first usage on line 493
The CADD scores (Combined Annotation Dependent Depletion scores) indicates the deleteriousness of a genetic variant. We have added the definition of CADD scores on its appearance in the methods section (p. 23).

11. For data availability, it would be useful if the authors could also make the individual height-adjusted summary stats for brain volume available for UK Biobank and ENIGMA and the height-adjusted summary stats for head circumference from Haworth et al.
We agree that our height-corrected summary statistics may be useful for other groups to use for future analyses and plan to make the height-corrected GWAS summary statistics available on our website. However, the GWAS results from ICV and head circumference were obtained from the ENIGMA consortium and the Max Planck Institute research group, for which redistribution of the results on our website is not allowed. The GWAS results of these samples are freely available on the website of the respective research groups.

We now mention the sharing of height-adjusted UKB summary statistics in the data availability section (in addition to sharing the meta-analysis results), and provide a short instruction of how our results can be reproduced by downloading the other summary statistics from their website (p. 29):

*“We have also made the height-corrected BV summary statistics in UKB available, yet data restrictions do not allow redistribution of the summary statistics of ICV and head circumference. To replicate our results, these summary statistics can be requested for download from the ENIGMA and Max Planck Institute websites (see **URLs**) The variant effect sizes can be subsequently corrected for height (summary statistics available from GWAS ATLAS; ID 3187) by using mtCOJO software.”*

12. Some of the journal volumes and page/article numbers listing in the references are incorrect or missing. For example, Savage et al. is listed as Nature Genet, 1, but should be Nature Genet, 50, 912–919. Haworth et al is missing the article number (357), as is Watanabe et al. (1826). Please check all references for completeness and accuracy.
We thank the reviewer for noticing these inconsistencies and have gone through our reference list; references have been corrected and updated where necessary.

13. In the Supplementary information lines 46-47, an r value is provided for those under 6 years of age, but not for the older age groups. Please could the r values for the older age groups be added?
We have now explicitly added the multiple r values for each of the three age groups as follows (Supplementary Information p. 4):
“The authors reported that HC was an excellent predictor for BV in children 6 years of age or younger (r=0.93). For the older age groups in their study, HC was somewhat less strongly predictive (17-42 years old, age not included: r=0.69) or only a significant predictor when also accounting for the subjects’ age (7-16 years old, age included: r=0.67).”

14. In supplementary figure 4, the coloring of the LD for each of the zoom plots are not displaying correctly. Would it also be possible to use dots to represent each SNP rather than boxes?
This was likely a consequence of the PDF formatting, we have replaced the pdf with the actual images of the figures to ensure visibility of the figure.

15. In supplementary table 1, the sample sizes for the studies within ENIGMA (n = 13,688) don’t quite add up to the total reported for ENIGMA (n = 11,373). The same is true for the HC study, although only differing by 3 participants. Please double check these numbers.
The reported sample sizes were the maximum number of participants in the meta-analysed cohorts, however due to differences in the availability of a SNP in all of the meta-analysed cohorts, the actual N varies per variant and is lower than the sample size. We agree that this is somewhat confusing as the numbers do not add up. We now report the largest per variant N of each cohort in supplementary table 1, which adds up to the total sample size of the meta-analysis. The full list of participating cohorts for each sample can be obtained through the supplementary tables of the Hibar et al., and Haworth et al. articles that report these GWAS studies. We have also added a note in supplementary table 1 where we state that the N represents the largest N reported for a variant in the GWAS summary statistics that we used, and that an overview of each of the individual cohort sample sizes can be retrieved from the original publication.

Reviewer #3 (Remarks to the Author):

The authors present a study examining the genetic coheritability of brain volume (BV) and intelligence (i.e., a measure of the extent to which the link between brain volume and intelligence can be explained by genetics). The authors proceed by considering a meta-analysis of the ENIGMA, UK Biobank and other consortia and by using the popular Linkage Disequilibrium Score Regression (LDSC) method for estimating genetic correlation in meta-analysis.

The authors report a genetic correlation between BV and intelligence of 0.21 (page 8, line 159). The authors also follow up with additional results such as genetic correlation between BV and other traits such as educational attainment, partitioned heritability in which GWAS are examined for enrichment in annotated areas of the genome, and discussion of involved genes.

The quality of the science in this paper is very high and the methodology is valid and well described. And the authors have replied extensively to concerns raised in a previous review, greatly improving the quality of the paper.

However, I view some of the points previously raised by reviewers as still unresolved.

Regarding comment 1 of Reviewer #3. The reviewer recommends that socioeconomic indicators be regressed out as a control. The authors respond “The main purpose of the current study is not to dissect the relation between socioeconomic factors and brain volume or intelligence. We believe this might be stigmatizing ...” I believe that it’s actually the opposite that is stigmatizing. It’s the failure to control for socioeconomic indicators that is stigmatizing. Imagine a SNP becomes correlated with a particular geographic region through genetic drift. Suppose that subjects living in this area have lower than average brain volume due to childhood malnutrition and also lower than average intelligence due to reduced access to education. This hypothetical SNP will appear as a

contribution to the genetic coheritability of BV and intelligence, as it covaries with geography. However, this contribution is irrelevant to questions of biology or basic science. Consideration of this SNP without "dissecting the relation between socioeconomic factors and brain volume and intelligence" may be stigmatizing.

Now the question about whether or not this is confounding the authors study. The authors have now included a version of their GWAS with 100 genetic PCs (discussed in their reply to comment 12 of Reviewer #2), and we can see there's not much difference in the Manhattan plots. That indicates no population stratification. However, this functions only as a most basic control for socioeconomic status. More work is required in order to adequately control for socioeconomic indicators.

Because of potential confounding effects of socioeconomic factors that are mentioned by reviewer 3, it was suggested by this reviewer and the editor that we correct the analyses for the Townsend deprivation index (TDI). We note that we already corrected the analyses in UKB for the Townsend deprivation index (TDI), exactly because we agree it is important to correct for potential socioeconomic factors captured by this index. TDI is a global index of socioeconomic status (Townsend et al., 1987) based on employment, home/car ownership and household overcrowding, which has been shown to correlate with individual health and deprivation measures (Adams et al., 2005), and morbidity and mortality (Smith et al., 2001). We agree that this was only mentioned briefly in the previous version and have now added a bit more rational on why we included TDI as a covariate.

We have made the following changes to the manuscript:

- In the main text, we mention the use of TDI as a covariate for socioeconomic status with a reference to aforementioned article that link TDI to individual level measures (p.4): *"GWAS analyses in UKB were corrected for the Townsend deprivation index (TDI), a measure that correlates with socioeconomic and health-related factors, as well as for age, sex, genotype array, assessment center, standing height and the first 10 genetic principal components."*
- We now mention in the methods section that TDI was included to adjust for confounding effects of socioeconomic factors (p. 21): *"We also include the Townsend deprivation index (TDI) as a covariate to correct for potential socioeconomic factors"*
- We have added a description and rational for using TDI as a measure of confounding in the supplementary methods section (p. 3): *"GWAS analyses in UKB were corrected for 10 genetic principal components (PC, see **Supplementary Results 3** for analyses corrected for 100 PCs), age, sex, genotype array, assessment center, height and the Townsend deprivation index (TDI) at recruitment. The TDI is an indicator of socioeconomic status (SES) and was calculated for each participant based on area of residency prior at enrollment into the UKB study. This measure is based on home and car ownership, employment and household overcrowding linked to postal code area. TDI is strongly associated with individual levels of deprivation[3] and health-related factors including self-reported health[4], life style factors (smoking[5], vegetable intake[6]), chronic illness[7], and all cause mortality[8]. We believe that inclusion of TDI as a covariate is the most appropriate correction for SES and health-related factors, since this measure has a correlational pattern with a wide variety of potential confounding factors, and because of its availability in all UKB participants."*

Regarding comment 8 of Reviewer #2, it's still unclear why genetic correlation between BV and intelligence is important to basic science, or how this work could lead to practical application.

We thank the reviewer for this opportunity to further discuss the rationale and implications of our research question. We have added this in the discussion section:

*"**DISCUSSION***

We studied the genetics of BV and report 14 novel loci and 270 novel genes for BV. The gene-findings converged into 18 significantly associated gene-sets related to cellular signaling, division, and growth, mostly appearing independent in conditional analyses.

We found a significant positive genetic correlation with intelligence, using previously published GWAS result[2] in line with the genetic correlation estimated from twin studies[1]. We then explored whether specific genes or gene-sets drive this genetic correlation, and identified 92 genes that are associated with both traits,. Of these 92, 32 genes were indicated as the most plausible ones explaining the shared genetic etiology . Several of these genes are involved in cell growth and differentiation pathways (e.g., *FOXO3, ERBB3, DDR* and *HBEGF,* **Fig. 4**). We also tested whether the set of 92 shared genes, had specific biological characteristics that would set them apart form the genes that were associated with either BV or intelligence. Except for an increased intolerance to LoF mutations, we did not find any other of the tested biological functions to be different We note that that the current set of genes associated with both traits, likely underestimates the true number of shared genes, as current sample sizes of especially the BV GWAS may limit the number of significantly associated genes.

The nature of the genetic overlap between intelligence BV remains largely unresolved. The large numbers of genes (>1,000) that well-powered GWAS are now able to identify[2,61,62] provide a starting point for understanding how genetic variation contributes to intelligence, yet it does not directly provide insight into the genetic relation with BV. Investigating genes that are related to both brain function (intelligence) and structure (BV), may provide further insight into the nature of the relation between intelligence and brain volume. We emphasize that in

addition to shared pathways and biological pleiotropy, some genes linked to intelligence may also be associated to BV through gene-environment correlation with factors that indirectly influence BV development[63].
In conclusion, we explored and refined the genetic architecture of BV, and identified and characterized a set of genes that likely drives this association. ”

Reviewer #4 (Remarks to the Author):

This is a revised version of a manuscript that investigates the specific genetic factors driving the well known phenotypic correlation between brain volume and IQ.
This study offers new information in this new meta-analysis of ~47K individuals in which 18 genomic loci (including 14 novel loci), are identified, implicating 343 genes (270 novel) and 18 biological pathways for brain volume. They identify 92 shared genes between BV and intelligence, which are mainly involved in signaling pathways regulating cell growth. The manuscript has a number of strengths, including some important followup analyses for understanding the possible mechanisms underlying these relationships, including examination of the pathways implicated, tissue and gene-expression analysis, Mendelian randomization analysis, and partitioning of heritability.
The authors have responded in detail to many of the previous detailed questions from the reviewers, and thus my remaining comments are minor /'big picture' comments.

-That there are 143 monogenic disorders caused by high-penetrance mutations in 89 of the 343 BV-implicated genes is an interesting finding, regarding the genetic architecture of determinants of brain volume. Notably, also the higher proportion of mutation intolerance for genes implicated in both BV and intelligence (vs either trait alone) is very interesting. How different is this from what would be expected by chance?
We thank the reviewer for this comment. We agree it would be of relevance to provide the number of genes that is expected based on the portion of genes involved GWAS and OMIM, assuming these proportions are independent. To address this, we have added to the manuscript:

- The main text of the manuscript now mentions the number of genes that would be expected based on the proportion of genes involved in our GWAS and in OMIM (p. 6 and p. 9-10).
- We have updated Fig. 3g, to also include the proportion of pLI > 0.9 and those being involved in developmental disorders of all genes (not included in BV, intelligence or both).

- The analysis of regional gene expression using the AHBA, which concludes that the set of overlapping genes for BV and intelligence is not preferentially expressed in any particular cortical region, seems a bit circular, given that the association is for two highly global traits, by definition. The motivation behind this particular analysis is unclear.
The aim of these analyses was to investigate whether the genetic correlation between BV and intelligence is driven by genes that are differentially expressed in certain brain area. We now clarified the motivation in the methods section of the text (p. 27): *“We investigated whether genes overlapping between BV and intelligence show different brain expression patterns than non-overlapping genes. This may provide evidence whether certain brain areas can explain the underlying genetic association between BV and intelligence.”*

We note that the main text already contained a motivation to these analyses (p. 10): *“Previous studies have sought to narrow down the link between intelligence and BV to specific subcortical regions, such as the caudate nuclei[9] or the thalamus[10]. If specific brain regions are related to intelligence, one might expect genes associated to intelligence (or BV) to be differentially expressed across regions”*. We did not further expand the clarification in the results section.

-Regarding the previous comment that there was not adequate control for socioeconomic indicators, the authors indicate that they did not wish to conduct 'stigmatizing analysis'. it is ironic that the measure of intelligence used here, Spearmans' g, was used to support the eugenics movement. This is just a comment, however, probably outside the scope of this manuscript. Nevertheless, a bit more thoughtful commentary about the role of gene-environment correlation in these findings would be welcome.

In response to a point by reviewer #3 concerning the rationale of our study, we have added a discussion section, where we also mention the possibility of gene-environment correlations as an explanation for the genetic overlap between BV and intelligence (p.11-13), see our response to reviewer #3 above.

REVIEWERS' COMMENTS:

Reviewer #2 (Remarks to the Author):

Reviewer comments:

Comment to Author:
The authors should be commended for producing a very readable and informative piece of research. I have reviewed this manuscript in two previous rounds of revision and I am satisfied that the authors have dealt with all but one of my previous comments (see comment 4 below). I have only a few minor comments which are detailed below using the line numbering in the pdf:

Comments

1. On line 99, the authors state "…19 risk loci were mapped…". Should this be 18 risk loci?
2. On line 103, I don't think it is quite clear enough what the differences is between "replicated", "not replicated" and "novel". Please could the authors elaborate slightly more in the text to make it clearer for readers?
3. On line 239, the authors use the phrase "…turned out negative". To avoid any misinterpretation (i.e. a negative interaction) this should be changed to reflect that no interaction was found.
4. On line 272, the authors state that the genetic correlations they observe are "in line with the genetic correlation estimated from twin studies". In the current response to reviewers they agreed not to compare these two calculations (reviewer 1, comment 5) and corrected it in that instance but have reintroduced it here in the discussion.
5. On line 278, typo: "…apart form…" should be "…apart from…"
6. On figure 3a and 3b the authors use different log scales for comparing the observed and expected. This is fine, but to aid the reader it would be useful to include in the figure legend what the solid horizontal line, solid diagonal line and dotted diagonal lines represent.

- David Howard

Reviewer #4 (Remarks to the Author):

I appreciate the thorough revision and consideration of this round of comments from the reviewers. As such, I have only very minor remaining comments.

p. 12 , line 271- set them apart *form* the genes (should be "from')

p 12, line 273-should be a period after the word 'different '
